# Integrated analysis of mRNA-seq and miRNA-seq reveals the advantage of polyploid *Solidago canadensis* in sexual reproduction

**DOI:** 10.1186/s12870-021-03240-x

**Published:** 2021-10-11

**Authors:** Miao Wu, Huiyuan Liu, Bingbing Li, Tao Zhu

**Affiliations:** College of Life Sciences and Engineering, Henan University of Urban Construction, Pingdingshan, 467041 Henan China

**Keywords:** Polyploidy, *Solidago canadensis*, Plant invasion, Sexual reproduction, Transcriptome, miRNA

## Abstract

**Background:**

The invasion of *Solidago canadensis* probably related to polyploidy, which may promotes its potential of sexual reproductive. *S. canadensis* as an invasive species which rapidly widespread through yield huge numbers of seed, but the mechanism remains unknown. To better understand the advantages of sexual reproduction in hexaploid *S. canadensis*, transcriptome and small RNA sequencing of diploid and hexaploid cytotypes in flower bud and fruit development stages were performed in this study.

**Results:**

The transcriptome analysis showed that in the flower bud stage, 29 DEGs were MADS-box related genes with 14 up-regulated and 15 down-regulated in hexaploid *S. canadensis*; 12 *SPL* genes were detected differentially expressed with 5 up-regulated and 7 down-regulated. In the fruit development stage, 26 MADS-box related genes with 20 up-regulated and 6 down-regulated in hexaploid *S. canadensis*; 5 *SPL* genes were all up-regulated; 28 seed storage protein related genes with 18 were up-regulated and 10 down-regulated. The weighted gene co-expression network analysis (WGCNA) identified 19 modules which consisted of co-expressed DEGs with functions such as sexual reproduction, secondary metabolism and transcription factors. Furthermore, we discovered 326 miRNAs with 67 known miRNAs and 259 novel miRNAs. Some of miRNAs, such as miR156, miR156a and miR156f, which target the sexual reproduction related genes.

**Conclusion:**

Our study provides a global view of the advantages of sexual reproduction in hexaploid *S. canadensis* based on the molecular mechanisms, which may promote hexaploid *S. canadensis* owing higher yield and fruit quality in the process of sexual reproduction and higher germination rate of seeds, and finally conductive to diffusion, faster propagation process and enhanced invasiveness.

**Supplementary Information:**

The online version contains supplementary material available at 10.1186/s12870-021-03240-x.

## Background

Biological invasion acted as a global threat to biodiversity [1]. Many invasive species pose a serious threat to local biodiversity, the invasion of alien plants will affect and simplify the new state of ecosystem [2]. The invasive plant show significantly negative impacts on the native ecosystems. Thus, plant invasion will ultimately affect human and society include reduction of agricultural production, obstacle of sustainable development and influence on human health [3]. Many studies have been dedicated to explore the mechanism of plant invasion [4–6]. At present, no common mechanism has been found to explain and predict that why some alien species rapidly expand beyond their local range and become dominant in non-native habitats [7, 8]. Thus, understanding the factors that support the rapid spread and successful growth in invasive species is necessary to design effective control strategies.

Reproduction as an essential role for invaders when they are introduced into a new habitat [9]. Thus, reproductive traits especially for the sexual reproduction generally have developed into important determinants of invasion [10, 11]. The advantages of sexual reproduction include the increased offspring diversity, reduced intraspecific competition and wider distribution potential for rapidly initiating new populations far from the parental plants [12]. Invasive plants have greater advantages in seed production capacity or seed size in the introduced range to speed up naturalization and spread [13]. Some invasive species spread rapidly via seed reproduction in the invasion process [11, 14]. Therefore, knowing the priority of sexual reproduction mechanism in the invasion success of exotic species is essential to understanding invasion strategies for invasive species.

Polyploid plants have been developed a series of drastic competitive traits. Polyploidy usually are morphological different to diploid [15]. This significant morphological variation will enhance plant vigor that may confer polyploids a pre-adaptive advantage, making them more likely to become invasive weeds than their diploid ancestors [16, 17]. Polyploid plant possessed a higher competitive advantage in the early stages of invasion due to the higher germination rate, faster growth and more robust seedlings. A greater sexual and vegetative reproduction ability may be helpful to establish and expand polyploid population [18]. Therefore, polyploidy might be one of the potential determinants in invasion success through a greater competitive ability than diploids [17, 19]. The comparative study of invasive species and native congeners is a common means to elucidate the evolution of competitiveness [20, 21], and many studies have revealed differences in the competitiveness of cytotypes in some alien invasive species [22–24]. However, a comprehensive overview of ploidy levels underlying molecular mechanism across the most invasive plant species is still lacking. Therefore, the studies of the difference of alien plant with different cytotypes from molecular basis levels will provide a better understanding of its molecular mechanisms about invasiveness for polyploids. Furthermore, such information may be essential for establishing the importance of ploidy in determining invasion success.

The whole genome duplication will lead to large-scale changes in the gene expression level [25–27]. These large scale changes are mostly including chromatin remodeling, methylation, and the production and function of small RNA [27–29]. MicroRNAs (miRNAs) are a class of small noncoding RNAs, which consisting of approximately 20-24 nucleotides in eukaryotes. In general, miRNA negatively regulate gene expression through complementarity to target mRNAs at the post-translational level [30, 31].

In recent years, small RNAs, especially miRNAs, have become important regulators of plant growth, development and stress response [32–34]. Some studies have shown that miRNAs play an important role in the sexual reproductive process of plants, which including the regulation of flowering time and flower development [34–37], and fruit ripening, seed yield and development [37–40]. In addition, the regulation function and expression pattern of miRNAs were changed and elucidated important molecular mechanisms in polyploid plant [41–43].

*Solidago canadensis* was a perennial herb with three cytotypes, diploid (2n = 2x = 18), tetraploid (2n = 4x = 36) and hexaploid (2n = 6x = 54), which has been introduced into many areas in the world from North American and has developed into invasive species including China [44]. Based on the different cytotypes, *S. canadensis* was also showed the different invasiveness ability. Polyploidy facilitate *S. canadensis* becoming competitive ability and promotes its successful formation of invasive species in China [22]. For example, polyploidization can promote *S. canadensis* to adapt in new environment [45]. The allelopathy potential of *S. canadensis* were enhanced by polyploidization, therefore, making the introduced polyploid *S. canadensis* more competitive than diploid, which helps its successful invasion [46]. Besides, reproductive traits of invasive plants are important determinant of their invasion. *S. canadensis* as an invasive plant can spread by sexual reproduction and asexual reproduction by rhizome. Studies suggested that sexual reproduction facilitates new *S. canadensis* populations establishment [44]. The advantage of invasiveness of *S. canadensis* has owing to the abilities to yield a large number of seeds, which is closely related to the number of inflorescences [47]. The molecular basis for the allelopathic metabolite synthesis has suggested that the altered related gene expression may enhanced invasive potential of polyploid *S. canadensis* [48]. While few studies fully clarified the molecular mechanism for the sexual reproduction in *S. canadensis*.

In this study, we explored the expression of reproduction related genes and miRNAs between hexaploid and diploid *S. canadensis*. Based on the gene and miRNA expression characteristics in the inflorescence of flower bud and fruit development stage between two cytotypes of *S. canadensis*, we elucidate the biological function of these genes and miRNAs. Furthermore, we combined the expression profile of gene with miRNA to construct miRNA-mRNA interaction network to explore the regulatory action of miRNAs. Our work will finally provided the theoretical basis for further exploration of the invasion mechanism of polyploid *S. canadensis*.

## Result

### Gene expression patterns in diploid and hexaploid cytotypes of *S. canadensis*

**Fig. 1:**
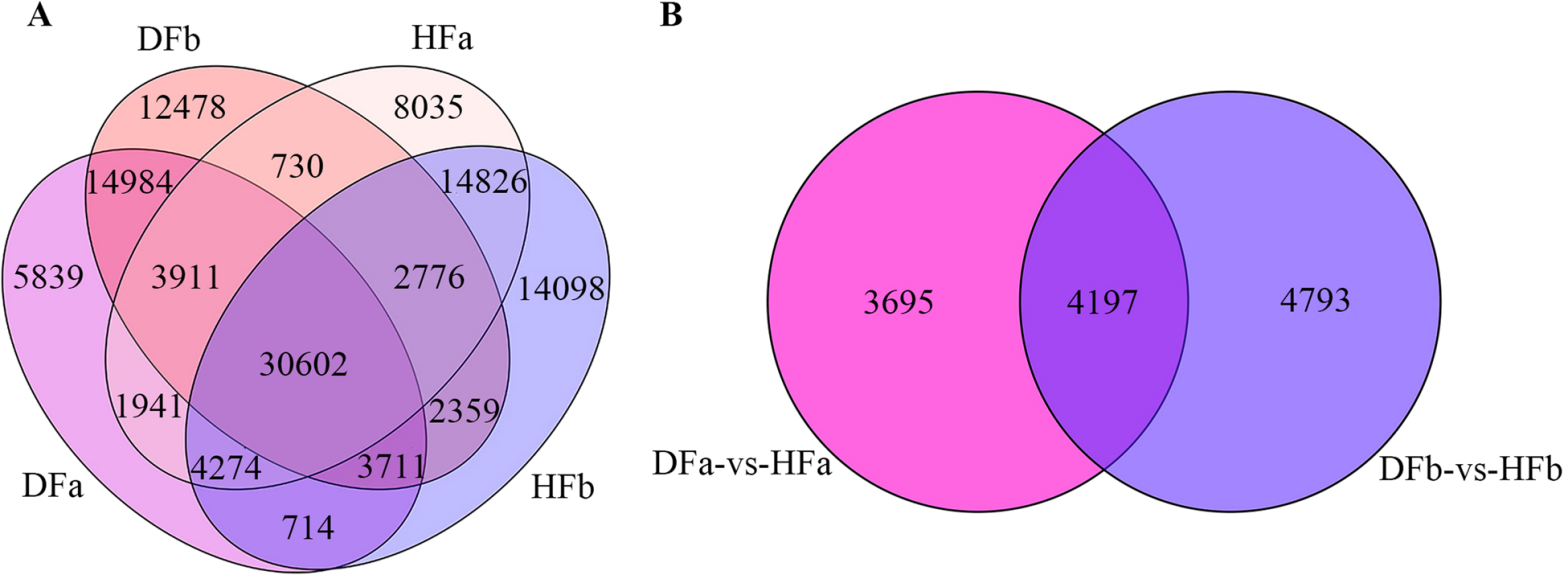
The distribution of expressed genes in each sample and differentially expressed genes between two cytotypes. **a** The distribution of expressed genes in the flower bud and fruit development stages of hexaploid (HFa and HFb) and diploid *S. canadensis* (DFa and DFb). **b** The distribution of differentially expressed genes at flower bud stage (DFa-vs-HFa) and fruit development stage (DFb-vs-HFb)

Total RNA was extracted from flower bud stage and fruit development in hexaploid (HFa and HFb) and diploid (DFa and DFb) samples with both of each group including 3 biological replicates. To explore the gene expression patterns in two cytotypes of *S. canadensis*, the raw data which obtained by RNA sequencing of the flower bud and fruit development stage in the hexaploid and diploid *S. canadensis* (a total of 12 samples) were filtered. The clean data for each sample was not less than 6 G with more than 99% Q20 bases and more than 96% Q30 bases were acquired (Additional file 1: Table S1). Based on the assembly of transcripts and the removal of low-abundance expressed genes, a total of 121,278 expressed genes were detected, of which 67,095, 73,360, 65,976 and 71,551 were detected in HFa, HFb, DFa and DFb respectively. Among all the detected genes, 30,602 genes were expressed in all four groups, 8035 genes were specifically expressed in HFa, 14,098 genes were specifically expressed in HFb, 5839 genes were specifically expressed in DFa, and 12,478 genes were specifically expressed in DFb (Fig. 1a). Furthermore, based on the alignment of differentially expressed genes (DEGs) between two cytotypes with the Gene Ontology (GO) and Kyoto Encyclopedia of Genes and Genomes (KEGG) databases, we found that 7892 genes were differentially expressed in the flower bud stage and 8990 genes in the fruit development stage. Among these differentially expressed genes, 4197 genes were detected in both flower bud and fruit development stage, 3695 genes were only detected in flower bud stage, while 4793 genes were only detected in fruit development stage (Fig. 1b).

### GO functional analysis of DEGs

**Fig. 2:**
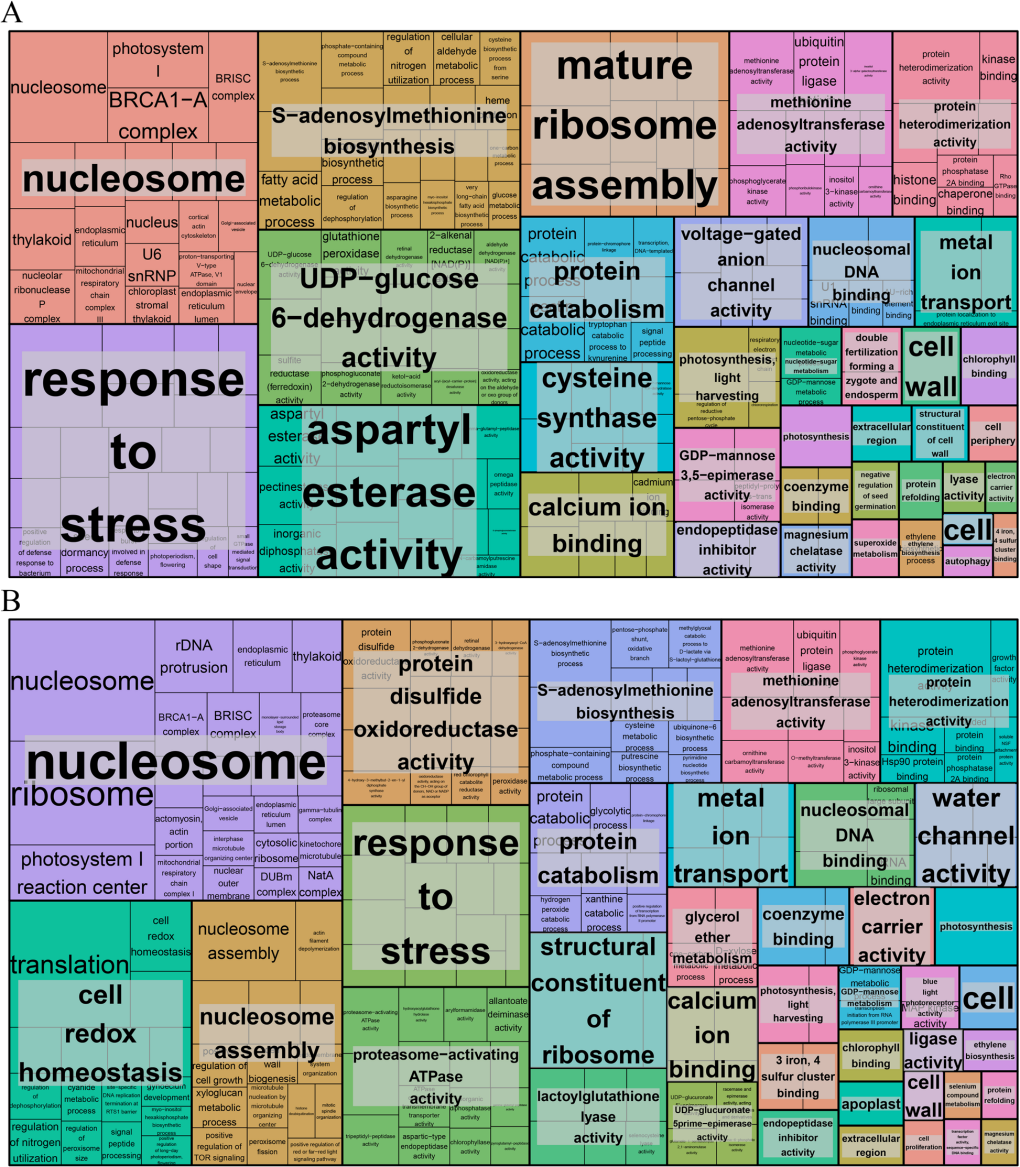
GO analysis of DEGs. **a** The DFa-vs-HFa comparision; **b** The DFb-vs-HFb comparision. Each rectangle indicates the enriched GO terms

To explore the insight into functional categories of the DEGs in the flower bud and fruit development stages, a GO classification was performed. In the flower bud stage, DEGs were distributed in 46 GO terms, which were mainly included in three categories: biology process, cellular component and molecular function. In the biology process category, most of DEGs were distributed in the subcategories of ‘cellular process’, ‘metabolic process’ and ‘biological regulation’ (Additional file 2: Fig. S1). In the cellular component, most of DEGs were distributed in the subcategories of ‘cell’, ‘cell part’ and ‘membrane’ (Additional file 2: Fig. S1). In the molecular function, most of DEGs were distributed in the subcategories of ‘catalytic activity’, ‘binding’ and ‘transporter activity’ (Additional file 2: Fig. S1). Furthermore, GO functional enrichment analysis was used to collect genes that play important roles in the flower bud stage. Based on the functionally de-redundant analysis of significantly enriched GO terms by REVIGO, we screened out many GO terms that are involved in biological functions such as ‘nucleosome’, ‘response to stress’, ‘mature ribosome assembly’, ‘protein catabolism’, ‘double fertilization forming a zygote and endosperm’ and ‘ethylene biosynthesis’ (Fig. 2a). The expression of genes which involved in these GO terms might be altered and played a vital role in the flower bud stage of hexaploid *S. canadensis*. In the fruit development stage, DEGs were distributed in 50 GO terms, which were also mainly included in three categories. In the biology process and cellular component category, most of DEGs were distributed similarly with flower bud stage (Additional file 3: Fig. S2). In the molecular function, most of DEGs were distributed in the subcategories of ‘catalytic activity’, ‘binding’ and ‘structural molecule activity’ (Additional file 3: Fig. S2). GO functional enrichment and functionally de-redundant analysis in the fruit development stage were also performed. Compared with the flower bud stage, there were no significant changes in the functional categories of DEGs in fruit development stage, while some changes were appeared in the enriched GO terms (Fig. 2b).

### Pathway functional analysis of DEGs

**Fig. 3:**
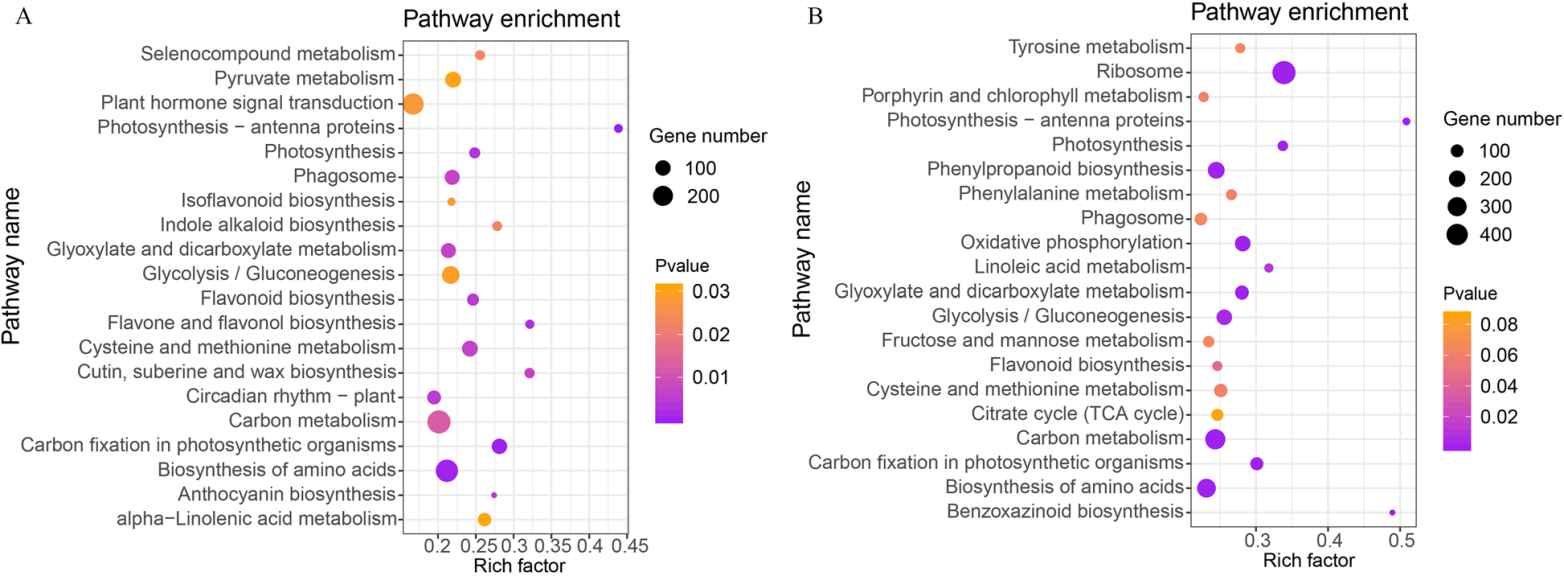
KEGG pathway enrichment analysis of DEGs. **a** The DFa-vs-HFa comparision; **b** The DFb-vs-HFb comparision. The Y-axis indicates the KEGG pathway, the X-axis indicates the rich factor. The bubble size indicates the number of DEGs of the pathway, and the colour bar indicates the q-value

The KEGG pathway classification and functional enrichment analysis were carried out to further identify the function of DEGs in flower bud and fruit development stages. The DEGs in the flower bud stage were mapped into 131 KEGG pathways, which most of genes were contained into the pathway of ‘Carbon metabolism’, ‘Ribosome’, ‘RNA transport’ and ‘Ubiquitin mediated proteolysis’ (Additional file 4 Table S2). The top 20 enriched pathways ranked by *Q*-value were mainly about ‘plant hormone signal transduction’, ‘circadian rhythm-plant’ ‘biosynthesis of amino acids’ and some of secondary metabolism related pathways such as ‘flavonoid biosynthesis’, ‘flavone and flavonol biosynthesis’ and ‘anthocyanin biosynthesis’ (Fig. 3a). In the fruit development stage, DEGs were also mapped into 131 KEGG pathways and most of genes were contained into the same pathways with flower bud stage (Additional file 5 Table S3). Some of the top 20 enriched pathways which ranked by *Q*-value were similar with the flower bud stage, such as ‘carbon metabolism’, ‘flavonoid biosynthesis’ and ‘biosynthesis of amino acids’. Some pathways, such as ‘ribosome’, ‘phenylpropanoid biosynthesis’ and ‘linoleic acid metabolism’ were specially collected (Fig. 3b). The DEGs which enriched in these pathways may play an important role in the reproductive development of hexaploid *S. canadensis*.

### Secondary metabolism related DEGs

**Fig. 4:**
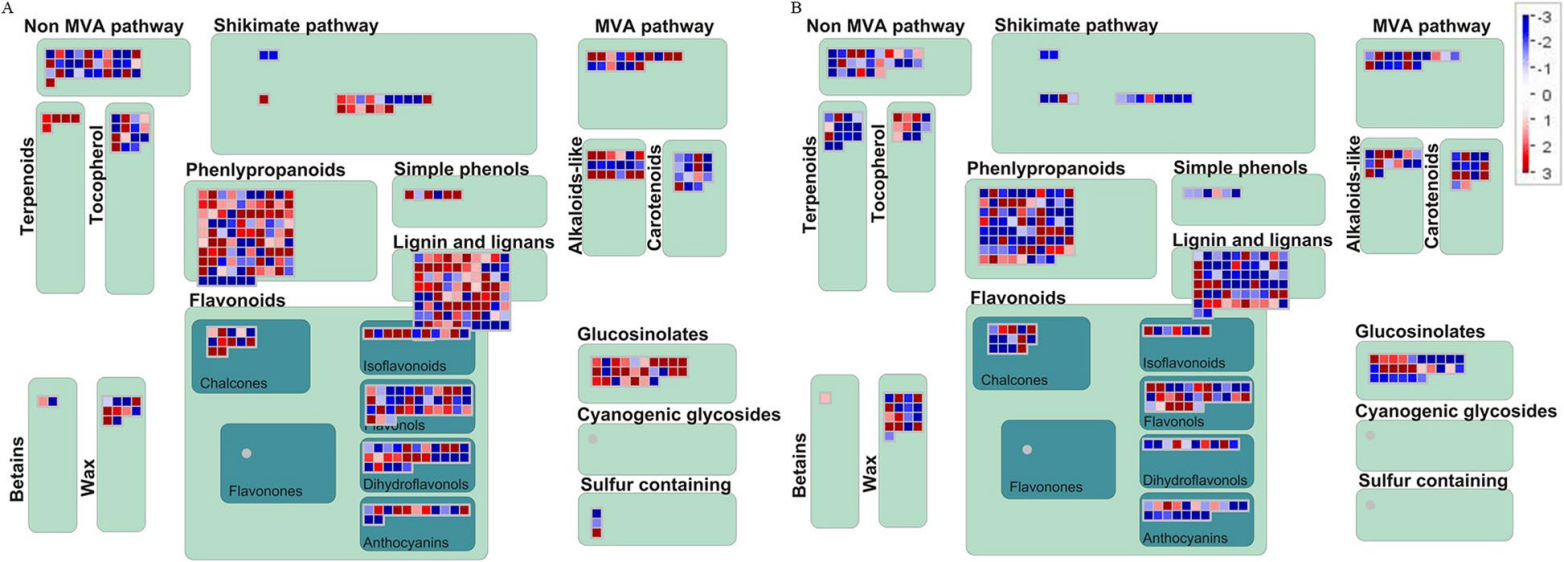
Mapman analysis of DEGs. **a** The DFa-vs-HFa comparision; **b** The DFb-vs-HFb comparision. The color bar indicates the normalized log_2_ transformed fold change value

According to the MapMan analysis, many DEGs which involved in secondary metabolite synthesis between two cytotypes of *S. canadensis* were collected in flower bud and fruit development stage (Fig. 4). In the flower bud stage, most of DEGs which mapped into the ‘phenlypropanoids’, ‘flavonoids’ and ‘terpenoids’ pathways were down-regulated in hexaploid *S. canadensis* (Fig. 4a). For example, the *PAL* (phenylalanine ammonia lyase), *C4H* (cinnamate 4-hydroxylase) and *4CL* (4-coumaroyl: CoA ligase) gene, which play a key role in ‘phenlypropanoids’ pathway, were down regulated (Additional file 6: Fig. S3). In addition, many DEGs related with terpenoid biosynthesis and metabolism were up-regulated. For example, some key enzyme genes which involve in the upstream of MVA pathway, such as *ACAA*, *HMGCR*, and *PMVK* genes were up regulated in hexaploid *S. canadensis* (Additional file 7: Fig. S4). In the fruit development stage, many DEGs mapped into the ‘phenlypropanoids’, ‘flavonoids’ and ‘terpenoids’ were down-regulated in hexaploid *S. canadensis* (Fig. 4b). While, in the phenylpropanoid biosynthesis pathway, *PAL*, *C4H*, *CHS* and *CHI* genes were up-regulated in hexaploid *S. canadensis* (Additional file 8: Fig. S5). In terpenoid synthesis related pathways, most of terpenoid biosynthesis related DEGs were down-regulated in hexaploid *S. canadensis* (Additional file 9: Fig. S6).

### Expression profiling of transcription factor (TF) genes

**Fig. 5:**
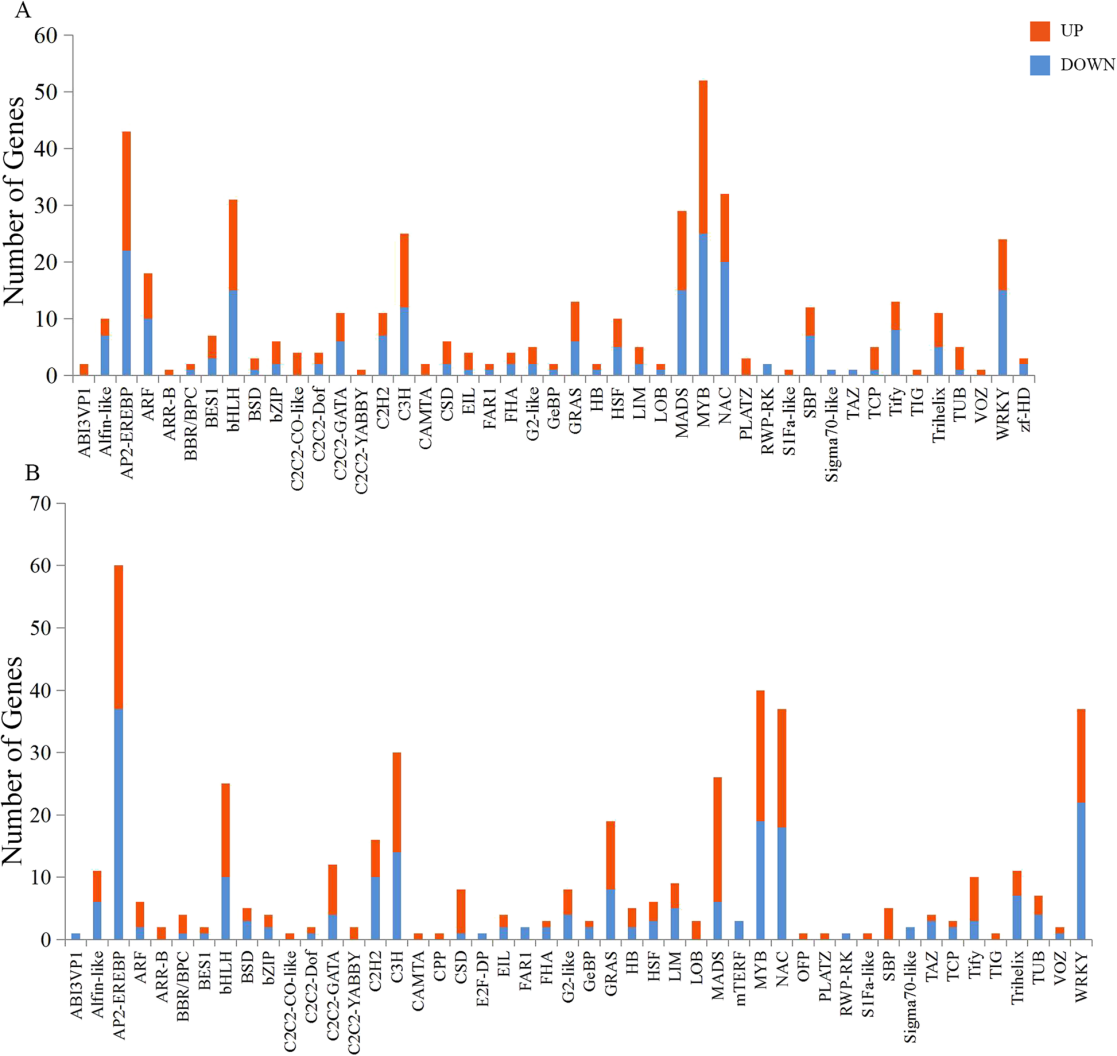
The number of differentially expressed TFs. **a** The DFa-vs-HFa comparision; **b** The DFb-vs-HFb comparision. The red colour indicates up-regulated TFs, the blue colour indicates down-regulated TFs

According to the differential expression analysis of TF genes between two cytotypes of *S. canadensis* in flower bud stage, 433 differentially expressed TF genes were obtained to be classified into 45 TF gene families. The most number of TF genes were *MYB* genes, with 27 were up-regulated and 25 were down regulated in hexaploid *S. canadensis*. As follows, 43 *AP2-EREBP* TF genes with 21 were up-regulated and 22 were down-regulated; 32 *NAC* TF genes with 12 were up-regulated and 20 were down-regulated; 31 *bHLH* TF genes with 16 were up-regulated and 15 were down-regulated; 29 *MADS* TF genes with 14 were up-regulated and 15 were down-regulated (Fig. 5a). In the fruit development stage, 448 differentially expressed TF genes were classified into 48 TF gene families. The most number of TF genes were *AP2-EREBP* genes with 23 were up-regulated and 37 were down-regulated in hexaploid *S. canadensis*. As follows were 40 *MYB* TF genes, of which 21 were up-regulated and 19 were down-regulated. In addition, other TF genes were also have different expression patterns compared with flower bud stage, such as *NAC*, *WRKY* and *C3H* TF genes (Fig. 5b). All of these differentially expressed TFs may play a major role in the growth and development in *S. canadensis*. Compared with diploid, the regulation mechanism in hexaploid *S. canadensis* may be altered.

### Sexual reproductive development related DEGs

Based on the pathway enrichment analysis, 79 DEGs were collected in plant-circadian rhythm pathway in the flower bud stage (Table 1). The expression pattern of these genes were various between two cytotypes *S. canadensis*. For example, there were 7 *PHYB* genes with 5 were up-regulated in hexaploid *S. canadensis*, and 2 down-regulated; 2 *GI* genes were up-regulated, a *CCA1* gene was down-regulated. Furthermore, 6 *LHY* genes with 3 up-regulated and 3 down-regulated; 7 *CO* genes with 5 up-regulated and 2 down-regulated. These DEGs may be involved in regulating the flowering process and flowering time in *S. canadensis*.

**Table 1:** Differentially expressed genes in the plant-circadian during flower bud stage

Gene Identifer	Gene Name	log_2_(Fold Change)	UP/DOWN
CCA1	CL7858.Contig1_All	−5.86	down
CDF1	CL8491.Contig1_All	3.19	up
CDF1	CL7233.Contig2_All	1.82	up
CDF1	CL8396.Contig2_All	−1.76	down
CDF1	CL7233.Contig3_All	−4.00	down
CHE	CL6494.Contig1_All	3.43	up
CHE	CL12062.Contig1_All	2.92	up
CHE	CL11296.Contig1_All	2.38	up
CHE	CL2760.Contig2_All	2.15	up
CHE	Unigene11750_All	− 2.98	down
CHS	CL657.Contig2_All	5.52	up
CHS	CL14895.Contig7_All	3.13	up
CHS	CL657.Contig6_All	−1.39	down
CHS	CL657.Contig5_All	−3.20	down
CHS	CL657.Contig1_All	−3.45	down
CHS	CL14895.Contig8_All	−3.49	down
CHS	CL657.Contig3_All	−4.55	down
CK2b	CL12797.Contig4_All	1.93	up
CK2b	CL3344.Contig2_All	−3.45	down
CK2α	Unigene40721_All	1.20	up
CK2α	CL5122.Contig1_All	−5.14	down
CK3α	CL5122.Contig2_All	−1.71	down
CK4α	CL5450.Contig1_All	1.13	up
CK5α	CL6117.Contig1_All	4.06	up
CK6α	CL6117.Contig2_All	1.37	up
CK7α	CL8572.Contig1_All	1.66	up
CK8α	CL8572.Contig2_All	−1.39	down
CO	CL9716.Contig2_All	5.29	up
CO	CL3756.Contig2_All	2.23	up
CO	Unigene56230_All	1.91	up
CO	CL15403.Contig1_All	1.90	up
CO	CL6412.Contig4_All	1.60	up
CO	CL4613.Contig1_All	−1.90	down
CO	CL6412.Contig3_All	−3.46	down
CRY	CL4897.Contig3_All	−1.80	down
GI	CL4000.Contig2_All	4.31	up
GI	CL4000.Contig1_All	2.91	up
HY5	Unigene48602_All	−1.94	down
HY5	CL16959.Contig2_All	−3.53	down
HY5	CL18750.Contig1_All	−4.64	down
HY5	CL19480.Contig4_All	1.65	up
HY5	CL19480.Contig7_All	−3.95	down
LHY	CL270.Contig10_All	1.73	up
LHY	CL7858.Contig2_All	1.09	up
LHY	Unigene40243_All	1.04	up
LHY	CL487.Contig3_All	−1.26	down
LHY	CL447.Contig5_All	−3.06	down
LHY	CL7858.Contig1_All	−5.86	down
PHYB	CL6361.Contig2_All	5.99	up
PHYB	CL12517.Contig3_All	5.03	up
PHYB	Unigene81014_All	4.04	up
PHYB	CL6361.Contig1_All	3.83	up
PHYB	CL9505.Contig4_All	2.79	up
PHYB	CL7348.Contig4_All	−1.71	down
PHYB	CL8822.Contig3_All	−4.64	down
PIF3	Unigene175_All	4.40	up
PIF3	CL6913.Contig1_All	4.20	up
PIF3	CL7457.Contig2_All	3.97	up
PIF3	CL1280.Contig2_All	2.88	up
PIF3	Unigene95348_All	2.74	up
PIF3	CL4218.Contig1_All	2.43	up
PIF3	CL11226.Contig3_All	2.05	up
PIF3	CL4218.Contig3_All	1.83	up
PIF3	Unigene33236_All	−1.04	down
PIF3	CL18653.Contig4_All	−2.40	down
PIF3	CL7457.Contig1_All	−3.51	down
PIF3	CL17590.Contig4_All	−4.22	down
PIF3	Unigene44150_All	−4.26	down
PIF3	CL1280.Contig3_All	−5.69	down
PRR5	CL6647.Contig2_All	2.51	up
PRR5	Unigene56230_All	1.91	up
PRR7	CL19525.Contig10_All	5.57	up
PRR7	CL19525.Contig13_All	5.04	up
PRR7	CL6647.Contig2_All	2.51	up
PRR7	CL2037.Contig3_All	1.67	up
PRR7	CL12994.Contig10_All	−3.03	down
PRR7	CL6647.Contig4_All	− 3.33	down
ZTL	CL14110.Contig2_All	4.68	up
ZTL	CL14110.Contig1_All	−2.82	down

We have identified 29 differentially expressed MADS-box related genes in the flower bud stage, of which 14 were up-regulated in hexaploid *S. canadensis* and 15 were down-regulated. Among them were mainly about *AGL* (*AGL11*, *AGL8* and *AGL9* etc.), *AP1* and *SEP1* genes (Table 2). In the fruit development stage, 26 differentially expressed MADS-box related genes were detected, of which 20 were up-regulated and 6 were down-regulated in hexaploid *S. canadensis* (Table 3). There were 16 genes co-differentially expressed between two stages, 13 genes were differentially expressed specially in the flower bud stage, and 10 genes were differentially expressed specially in fruit development stage. According to these differentially expressed genes, which belong to *MADS* TF gene families, we inferred that hexaploid *S. canadensis* may be different with diploid in flower morphogenesis, fruit ripening and development.

**Table 2:** Differentially expressed MADS-box genes in the flower bud stage

Gene Identifer	Gene Name	log_2_(Fold Change)	UP/DOWN
AGL11	CL12154.Contig2_All	5.09	up
AGL11	CL12154.Contig1_All	−2.00	down
AGL11	CL12154.Contig3_All	−2.55	down
AGL12	CL10755.Contig4_All	−1.36	down
AGL16	Unigene90070_All	−4.60	down
AGL65	CL14171.Contig1_All	4.83	up
AGL8	Unigene24590_All	4.53	up
AGL8	Unigene7682_All	3.03	up
AGL8	Unigene10808_All	2.36	up
AGL8	Unigene15863_All	−3.91	down
AGL9	CL6383.Contig3_All	8.90	up
AGL9	CL6383.Contig7_All	2.19	up
AP1	CL8240.Contig1_All	7.60	up
AP1	Unigene25953_All	1.69	up
AP1	CL18395.Contig1_All	1.21	up
AP1	CL8240.Contig3_All	−5.68	down
EJ2	CL12001.Contig1_All	1.16	up
MADS16	Unigene12177_All	−3.64	down
MADS6	CL6559.Contig3_All	−3.35	down
PHE1	Unigene56591_All	−1.38	down
PMADS1	CL2428.Contig2_All	−4.07	down
PMADS1	CL13463.Contig1_All	−5.57	down
PMADS2	Unigene40486_All	6.87	up
PMADS2	CL915.Contig7_All	1.55	up
PMADS2	CL915.Contig6_All	−1.77	down
PMADS2	Unigene40485_All	−2.44	down
SEP1	CL1712.Contig5_All	1.38	up
SEP1	CL1712.Contig17_All	−2.44	down
SEP1	Unigene45_All	−3.70	down

**Table 3:** Differentially expressed MADS-box genes in the fruit development stage

Gene Identifer	Gene Name	log_2_(Fold Change)	UP/DOWN
AG2	CL9917.Contig5_All	1.55	up
AG2	CL9917.Contig4_All	1.19	up
AGL11	CL12154.Contig2_All	8.96	up
AGL11	CL12154.Contig1_All	2.91	up
AGL65	CL14171.Contig1_All	5.76	up
AGL8	Unigene10808_All	5.30	up
AGL8	CL1694.Contig5_All	−3.09	down
AGL9	CL14513.Contig1_All	3.86	up
AGL9	CL6383.Contig9_All	3.42	up
AP1	CL8240.Contig1_All	6.18	up
AP1	Unigene25953_All	2.44	up
AP1	CL18395.Contig1_All	1.51	up
AP1	CL8240.Contig3_All	−1.56	down
DEFA	Unigene26195_All	2.89	up
EJ2	CL12001.Contig1_All	1.96	up
MADS15	CL4864.Contig8_All	4.17	up
MADS15	CL4864.Contig1_All	3.93	up
MADS32	Unigene56483_All	−5.22	down
MADS5	CL10755.Contig4_All	3.40	up
MADS6	CL6559.Contig3_All	−2.09	down
PHE1	Unigene56591_All	6.34	up
PMADS2	CL915.Contig7_All	1.36	up
PMADS2	CL915.Contig6_All	−2.07	down
SEP1	CL1712.Contig5_All	1.68	up
SEP1	CL1712.Contig11_All	1.17	up
SEP1	CL1712.Contig17_All	−1.79	down

Besides, 12 differentially expressed *SPL* genes were detected in flower bud stage, 5 of which were up-regulated and 7 down-regulated in hexaploid *S. canadensis*. In the fruit development stage, the detected DEGs with 5 *SPL* genes were up-regulated. In addition, 28 seed storage protein related genes were also detected in fruit development stage, 18 of which were up-regulated and 10 down-regulated in hexaploid. These genes played an important role in improving seed yield and quality. Therefore, the increased expression of these genes may play an important role of sexual reproduction in hexaploid *S. canadensis*.

### Gene expression network analysis

**Fig. 6:**
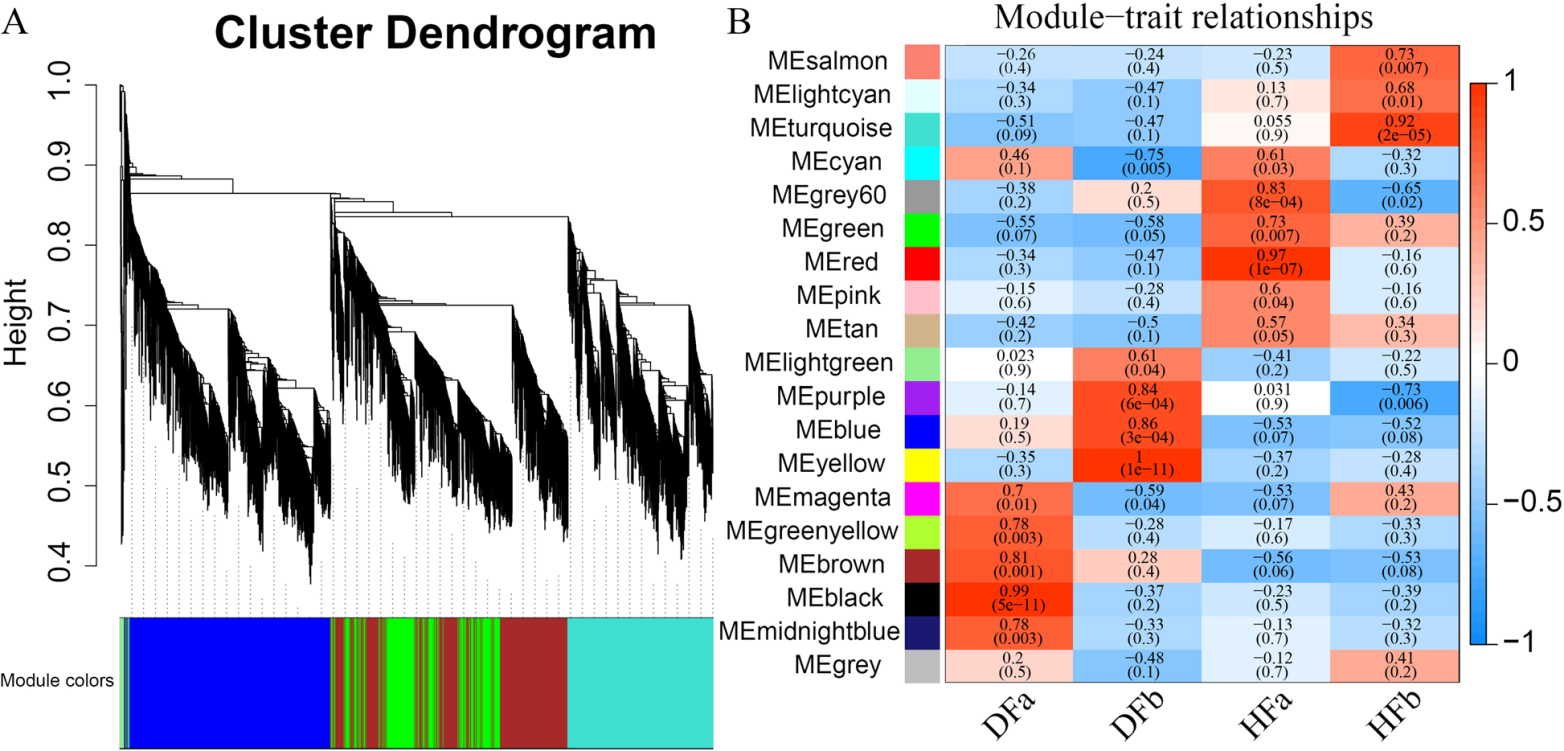
WGCNA analysis. **a** Clustering dendrogram of genes, with dissimilarity based on topological overlap, together with assigned merged module colors and theory final module colors. **b** Module–trait associations. The colour bar indicates the correlation of each module with each sample. Each cell contains the corresponding correlation and *p*-value

A total of 12,685 DEGs from two comparison groups in flower bud and fruit development stage were analyzed by the weighted gene co-expression network analysis (WGCNA). We identified 19 gene expression modules, which was classified the number of gene from 30 to 2484 (Fig. 6a). Based on the correlation analysis of the module and trait, many co-regulatory modules were collected (correlation coefficient > 0.6, *p* < 0.05) (Fig. 6b).

**Fig. 7:**
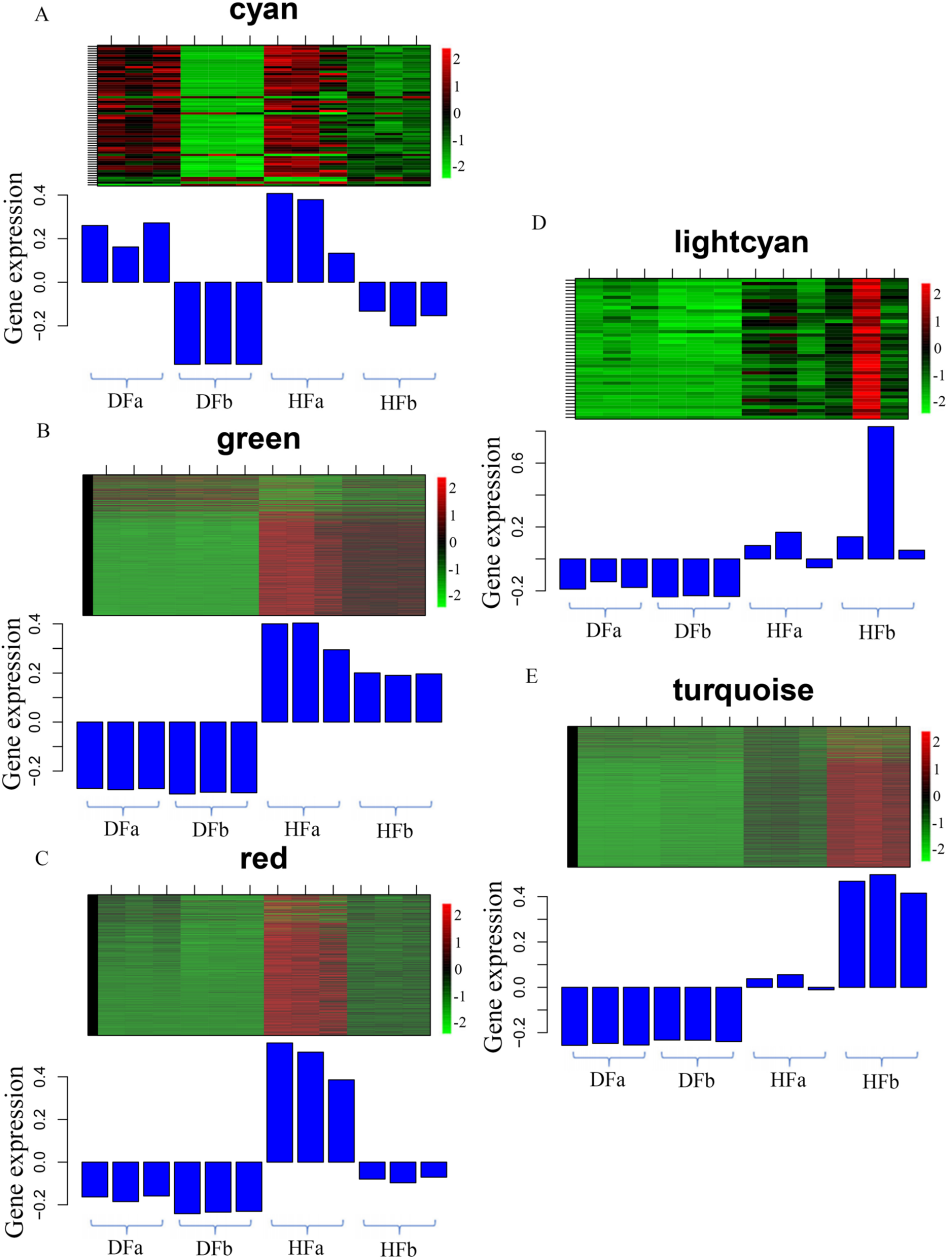
The heatmap of gene expression level in all samples of selected WGCNA modules. The colour bar indicates the relative expression of module genes, red denotes up-regulation, green denotes down-regulation

**Fig. 8:**
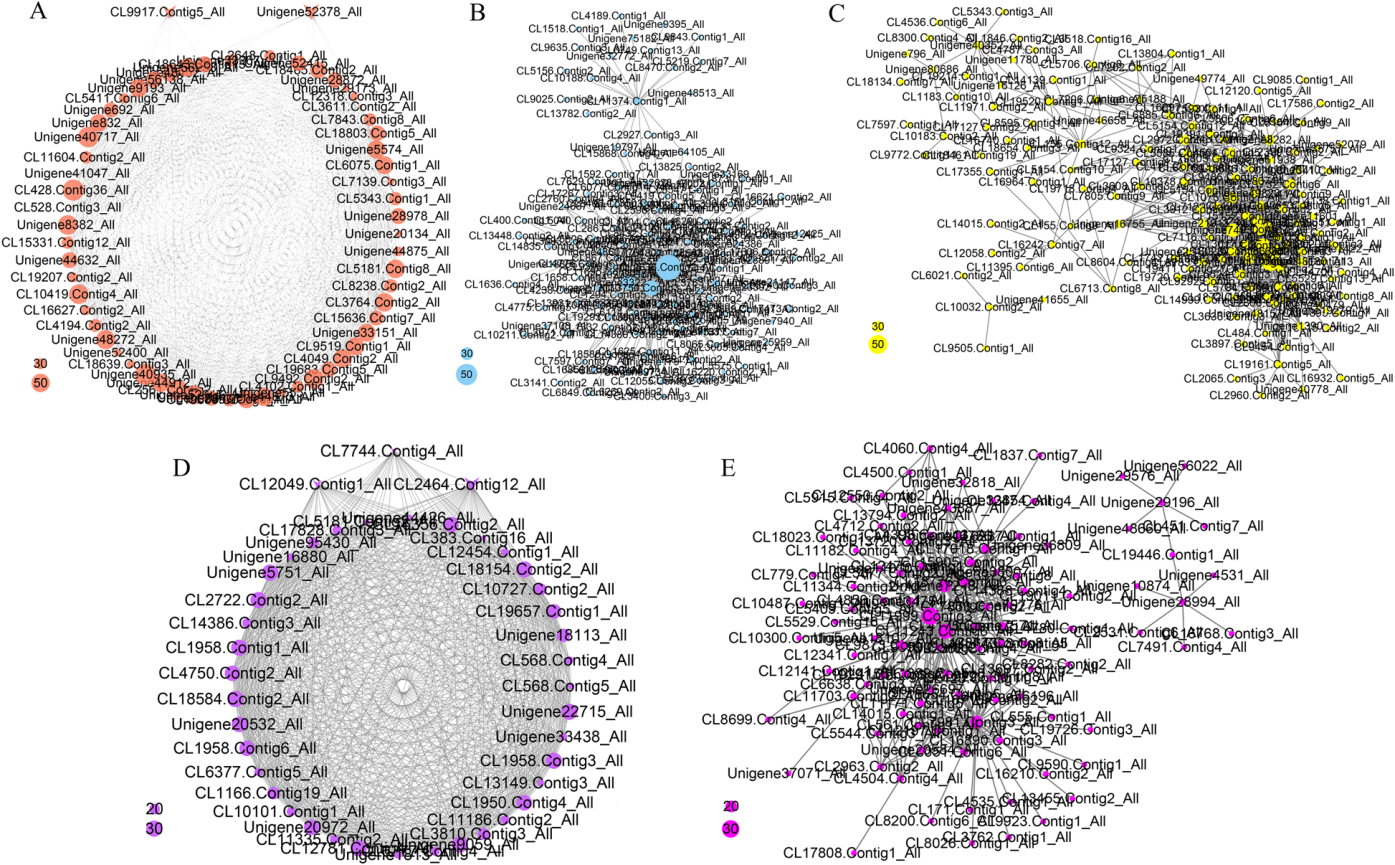
Interaction analyses of the selected modules. The bubble size indicates connect degree of each gene

In the HFa group, four modules with cyan, grey60, green and red were collected. To examine the transcript profiles of these modules, we depicted the heatmaps of genes across all samples. The cyan module with 61 genes were collected, most of which were up-regulated in DFa group and HFa group and down-regulated in DFb and HFb group (Fig. 7a). The network analysis showed that many of functional genes were included in this module (Fig. 8a), such as starch synthase gene (CL15636.Contig7_All), histone H3 gene (CL18639.Contig3_All, Unigene44875_All), histone H2A gene (CL19683.Contig1_All, CL19683.Contig5_All), histone H2B gene (CL8238.Contig2_All), auxin-responsive protein gene (CL7139.Contig3_All), gibberellin receptor *GID1* gene (Unigene56138_All), *MADS-box* TF gene (CL9917.Contig5_All), *MYB* TF gene (Unigene52378_All) and FLOWERING LOCUS T gene (Unigene832_All). In the green module, 1337 genes were collected, most of these genes were up-regulated in HFa and HFb group and down-regulated in DFa and DFb group (Fig. 7b). The network representation of 150 genes with WGCNA edge weight > 0.46 for further analyses (Fig. 8b), which included ubiquitin related genes (CL2636.Contig2_All, Unigene17130_All, CL2880.Contig5_All, CL4786.Contig3_All, CL1625.Contig11_All, CL1677.Contig2_All and CL13448.Contig2_All), histone related genes (CL1658.Contig4_All and Unigene37108_All), translation initiation factor genes (Unigene75377_All, CL17267.Contig3_All, CL17350.Contig2_All, Unigene28891_All and CL16220.Contig2_All) and some TF genes such as *MYB* gene (CL17002.Contig1_All), *SBP* gene (CL3869.Contig1_All) and *TCP* gene (CL2760.Contig4_All). In the red module, 1077 genes were collected, most of these genes were up-regulated in HFa group and down-regulated in DFa, DFb and HFb group (Fig. 7c). The network representation of 154 genes with WGCNA edge weight > 0.47 were used for further analyses (Fig. 8c), which included flavone and flavonol biosynthesis genes (CL11.Contig1_All and CL11.Contig2_All), ubiquitin related genes (CL19520.Contig1_All, Unigene17221_All, CL6885.Contig6_All, CL1548.Contig2_All, CL1203.Contig2_All, CL12120.Contig5_All, CL7593.Contig1_All and CL13587.Contig1_All), *MADS-box* TF gene (CL8783.Contig1_All, Unigene24590_All), *ARF* gene (Unigene25133_All), *CHS* gene (CL14895.Contig7_All) and *SBP* gene (CL2806.Contig1_All).

In the HFb group, three modules with salmon, lightcyan, and turquoise were collected. In the lightcyan module, 41 genes were collected, most of these genes were down-regulated in DFa and DFb group, and up-regulated in HFa and HFb group (Fig. 7d). The network analysis showed that some TF genes and secondary metabolite synthesis related genes were included in this module (Fig. 8d), such as cinnamyl-alcohol dehydrogenase (*CAD*) gene (Unigene18113_All and Unigene5751_All), flavonol synthase (*FLS*) gene (CL6377.Contig4_All), carotenoid cleavage dioxygenase (*CCD*) gene (CL11186.Contig2_All), *Tify* (CL2464.Contig12_All) and *MYB* (CL12049.Contig1_All) gene. In the turquoise module, 2484 genes were collected, most of these genes were up-regulated in HFa and HFb group, and down-regulated in DFa and DFb group (Fig. 7e). The network representation of 102 genes with WGCNA edge weight > 0.45 were used for further analyses (Fig. 8e), which included ubiquitin related genes (CL15905.Contig2_All, CL5915.Contig4_All and CL779.Contig7_All), *4CL* gene (Unigene28994_All), calcium-dependent protein kinase gene (Unigene26697_All), *FLS* gene (CL11344.Contig2_All) and germacrene D synthase gene (CL16768.Contig3_All). These collected genes may be vital for the regulation of sexual reproduction, metabolism and hormone signal transduction related processes in hexaploid *S. canadensis*.

### Small RNA profiling in diploid and hexaploid cytotypes of *S. canadensis*

Despite the numerous mRNAs involved in sexual reproduction, miRNAs, which, regulate mRNA expressions at the post-transcriptional level, also play vital roles in floral development. To investigate the small RNAs component and the dynamic changes of miRNAs between two cytotypes of *S. canadensis*, twelve small RNA libraries were generated from the flower bud and fruit development stage samples. After the deep sequencing, and removing low quality reads, adaptor, insert and poly (A) contaminations, average about 25.2, 26.7, 27.2 and 27.1 million clean reads were obtained in three replicate samples of DFa, DFb, HFa and HFb respectively (Additional file 10: Table S4). The length of distributions of miRNAs were range from 18 to 30 nucleotides, and the lengths of miRNAs in all samples were concentrated between 21 and 24 nt (Additional file 11: Fig. S7).

**Fig. 9:**
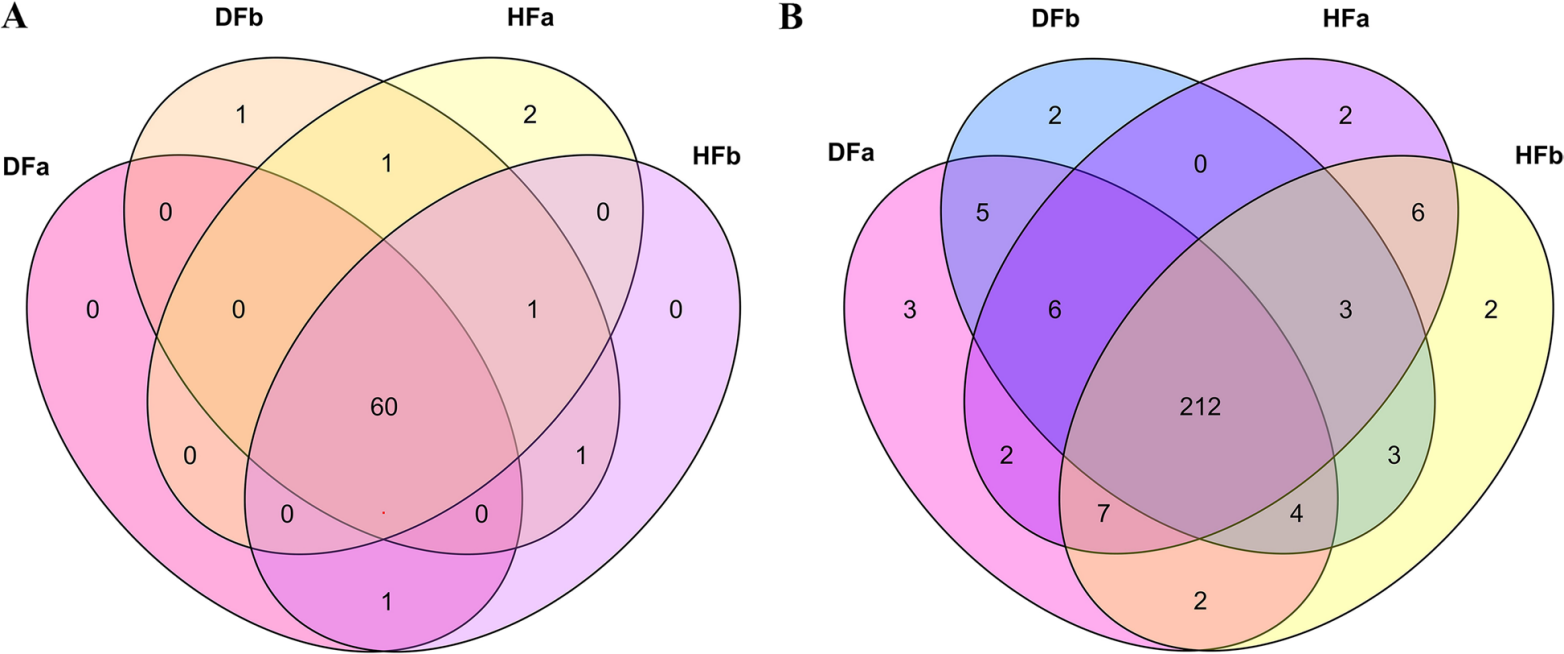
The distribution of expressed miRNAs in each sample. **a** The distribution of known miRNAs. **b** The distribution of novel miRNAs

A total 67 known miRNAs were finally identified in the flower bud and fruit development stage of *S. canadensis*, of which 61 were identified in DFa, 64 were identified in DFb and HFa, 63 were identified in HFb. There were 60 miRNAs in all libraries, 2 specifically expressed in HFa, and 1 specifically expressed in DFb (Fig. 9a). In addition, based on the structure of miRNAs, 259 novel miRNAs were predicted, of which, 241 were predicted in DFa, 235 in DFb, 238 in HFa and 239 in HFb. There were 212 novel miRNAs expressed in all libraries, 2 specifically expressed in DFb, HFa and HFb respectively, and 3 specifically expressed in DFa (Fig. 9b).

### Differential expression analysis of miRNAs and target prediction of miRNAs

**Fig. 10:**
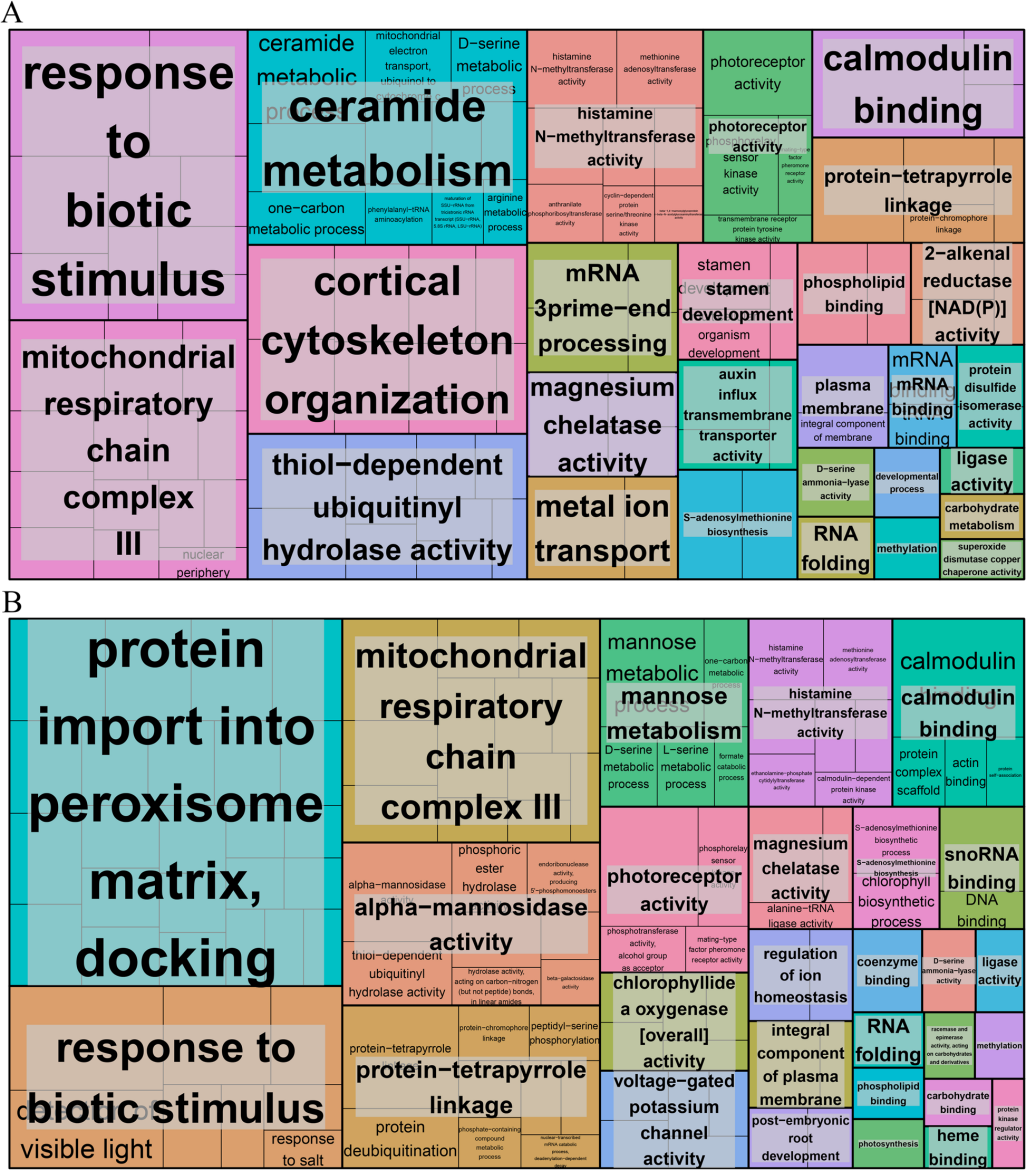
GO analysis of target genes of differentially expressed miRNAs. **a** The DFa-vs-HFa comparision; **b** The DFb-vs-HFb comparision

Based on differential expression analysis of miRNAs, 114 differentially expressed miRNAs (DEMs) were detected in the flower bud stage, of which 52 were up-regulated and 62 down-regulated in hexaploid *S. canadensis*. There were 3365 genes putatively targeted by multiple miRNAs in the flower bud stage. The GO functional classification revealed that these target genes were distributed in 43 GO terms. In the biology process category, most of DEGs were distributed in the subcategories of ‘cellular process’, ‘metabolic process’ and ‘biological regulation’ (Additional file 12: Fig. S8). Based on the GO enrichment analysis of these target genes and functional de-redundant analysis of the enriched GO terms, we found that many target genes were involved in some biological functions such as ‘response to biotic stimulus’, ‘thiol−dependent ubiquitinyl hydrolase activity’, ‘stamen development’ and ‘auxin influx transmembrane transporter activity’ (Fig. 10a).

Furthermore, 163 DEMs were detected in the fruit development stage, of which 109 were up-regulated and 54 down-regulated. There were 4003 genes putatively targeted by multiple miRNAs in the fruit development stage. These target genes were also distributed in 43 GO terms and most of genes distributed into the same GO terms with flower stage (Additional file 13: Fig. S9). The GO enrichment analysis and functional de-redundant analysis revealed that many target genes were involved in ‘response to biotic stimulus’, ‘histamine N-methyltransferase activity’ and ‘coenzyme binding’ (Fig. 10b).

### Visualization of miRNA-mRNA interaction network

**Fig. 11:**
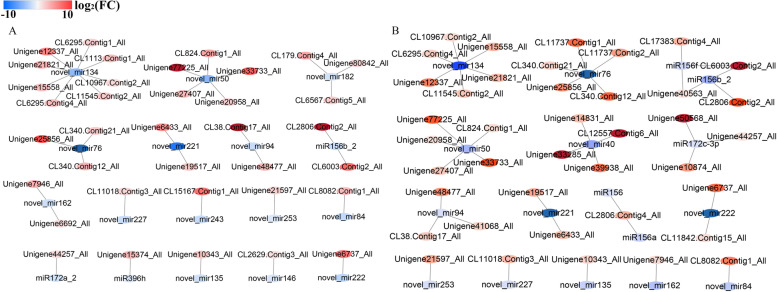
The interaction network of miRNAs with target genes. **a** The DFa-vs-HFa comparision; **b** The DFb-vs-HFb comparision

Based on the negative regulatory role of miRNA in the process of mRNA expression, in this study, the interaction network between miRNA and target genes in the flower bud and fruit development stage of *S. canadensis* were constructed. In the flower bud stage, 36 DEMs were detected to negatively regulate 383 target DEGs, of which 17 down-regulated miRNAs negatively regulate 36 target genes (Fig. 11a). In the fruit development stage, 56 DEMs were detected to negatively regulate 490 target DEGs, of which 17 down-regulated miRNAs negatively regulate 40 target genes (Fig. 11b). These target genes include *SPL* genes which involved in promoting growth and development, regulating flowering time and seed yield, TF genes and some enzyme-related genes. In the flower bud stage, some miRNAs were putative to target functional genes, such as miR156b_2 targeted with *SPL12* (CL2806.Contig2_All) and *SPL13A* (CL6003.Contig2_All) gene, miR172a_2 targeted with *ERF* (Unigene44257_All) TF genes, novel_mir50 targeted with *MYB* (CL824.Contig1_All) and zinc-finger protein (Unigene77225_All) TF gene. In the fruit development stage, some miRNAs were putative to target the same functional genes with that of flower bud stage, furthermore, these miRNAs were also have specific regulation effects. For example, except for targeting *SPL12* and *SPL13* genes, miR156 also targeted with *SPL10* (Unigene40563_All) gene. In addition, the specific differentially expressed miR156 and miR156a during this period targeted with *SPL12* (CL2806.Contig4_All) gene, miR156f targeted with *SPL10* (Unigene40563_All), *SPL13B* (CL6003.Contig2_All) and *SPL18* (CL17383.Contig4_All) gene. These DEMs may play a vital role in the flower bud and fruit development stage in hexaploid *S. canadensis*, and these miRNAs have differences regulation in the different stages.

### Confirmation the expression profile data of mRNAs and miRNAs by the quantitative real-time PCR (qRT-PCR)

To verify the accuracy of the expression of mRNA and miRNA obtained from mRNA-seq and miRNA-seq, we randomly selected 12 genes (Additional file 14: Fig. S10) and 7 miRNAs (Additional file 15: Fig. S11) for the qRT-PCR analyses. The relative expression levels of selected genes and miRNAs in each groups, which determined by qRT-PCR, were consistent with RNA-seq. The primer sequences used in this study were listed in the Additional file 16: Table S5.

## Discussion

*S. canadensis* spread rapidly in the invasion area owing to its great capacity for sexual reproduction [47]. Hexaploid *S. canadensis* have developed into an invasive plant may related to polyploidization [17], which induced the gene expression level alteration [48]. This study depicted mRNA and miRNA integrated analyses in the flower bud and fruit development stage of the *S. canadensis*. The results showed that hexaploid *S. canadensis* has significant differences in the regulation of gene expression related to hormone signal transduction, reproductive development and the synthesis of secondary metabolites. These differentially expressed genes in hexaploid *S. canadensis* may provide a certain advantage in sexual reproduction, and lay a foundation for its rapid spread and formation of invasive plants.

### Plant circadian rhythm related genes may affect the growth and development in hexaploid *S. canadensis*

Plants have capacities to adapt its survival environment by its internal circadian rhythm and regulate a series of physiological processes such as photoperiod, flowering time, hormone signal transduction, plant growth and metabolism, and response to biological and abiotic stresses. Studies have shown that CIRCADIAN CLOCK ASSOCIATED 1 (CCA1) protein can inhibit the expression of phytochrome B (PHYB) activation-tagged suppressor 1 (*BAS1*) gene to regulate the synthesis of brassinosteroids [49]. Cryptochrome 1 (*CRY1*) and *phyB* genes can directly interacted with auxin response factor (*ARF*) gene to regulate auxin signaling pathway [50]. The circadian rhythm system will affect flowering time in plants by sensing altered surrounding environment conditions (such as light and temperature) [51, 52]. *GI* and *CO* act as the key genes in the photoperiodic pathway, besides, the interaction of *phyA*, *CRY1* and *CRY2* genes will affected the expression of *GI* gene, which promote the expression of *CO* gene. The *CO* gene coding proteins which activate the *FT* gene, and then affected the expression of *LFY* and *AP1* genes. These genes constructed a series of complex networks that will eventually promote the floral organs development. Polyploidy can induced a series of related gene expression regulation changes, and then significantly affected the plant morphological vigor [53]. In this study, most of related genes were differentially expressed in flower bud stage. The key genes with a large number of differentially expressed genes, such as *phyB* and *PIF3*, were up-regulated in hexaploid. They may play an important role in the photoperiod signaling and downstream regulation of hexaploid. In addition, *PRR7*, *PRR5* and *CO* genes involved in flower development were also up-regulated in hexaploid. These genes may play a certain role in the growth and development of hexaploid *S. canadensis*, and the advantages in reproductive level.

### Flower and fruit development related genes may promote the reproductive process of hexaploid *S. canadensis*

*MADS-box* genes are widely distributed in plants and play an important role in the regulation of growth and development, particularly in the development of floral organs, flowering time regulation and fruit development and ripeness in angiosperms [54–56]. In Arabidopsis, *AGL23* gene participate in the regulation of the development of female gametophytes and the formation of organelles during embryonic development [57]; *AGL61* cooperated with *AGL80* gene to participate in the differentiation of female gametophytes [58]; *AGL62* gene can be involved in the formation of endosperm cells; *SEP* gene involved in the formation of floral organs [59]; *PHE1* gene plays an important role in the seed development and nutrient storage [60]; *AGL61* and *AGL80* genes were also involved in the development of central cells and endosperm [61, 62]. In addition, *AP1* gene will interferes with the specification of floral organs from common primordia into floral organs, and the true conversion of flowers into inflorescences in legumes [63]. *MADS1* and *MADS7* genes in orchids play a role in the development of stamens and ovary [64]. In this study, many *MADS* genes were differentially expressed between two cytotypes of *S. canadensis* in the flower bud and fruit development stage. In the flower bud stage, most of the up-regulated genes were *AGL8* and *AP1* genes, while some *AGL* genes, such as *AGL12* and *AGL16* were down-regulated in hexaploid *S. canadensis*. These different expression patterns in flower bud stage indicate that there were different regulatory modes between two cytotypes, and may be a certain bias in functional selection, which may causing in the formation and development of flowers. In fruit stage, most of *MADS-box*-related genes were up-regulated in hexaploid *S. canadensis*, which indicates that hexaploid *S. canadensis* may have a significant effect compared with diploid during fruit formation and development. The number of differentially expressed *AGL8* and *PMADS2* genes was decreased when compared with flower bud stage, while the number of genes such as *AP1*, *AGL9* and *AGL65* was not altered. This indicated that these related genes may have a temporal and spatial bias among the flower bud and fruit development stage in *S. canadensis*. For example, *AGL8* and *PMADS2* genes may play a more important role in the flower bud stage compared with the fruit development stage. While, some genes such as *AG2*, *MADS15*, *AGL11*, *PHE1*, and *SEP1* were detected up-regulated in hexaploid fruit development stage. Some of these genes were specifically and differentially expressed in the hexaploid *S. canadensis*, such as *AG2* and *MADS15*, which may be vital to the development of the fruit. In addition, plant seeds accumulate a large amount of storage protein to improve seed quality during maturation. In this study, a large number of seed storage protein genes were differentially expressed between two cytotypes and most of them were up-regulated in hexaploid *S. canadensis*, which was likely to improve seed quality and provide material basis for higher germination efficiency after dissemination.

### miRNA may play a vital role in the reproductive development of hexaploid *S. canadensis*

MiRNAs in plants play an important role in various regulatory processes such as growth, development, and stress resistance. Altered the regulation of miRNAs with target genes may lead to phenotypic changes in plants. OsmiR156 directed regulate the expression of *SPL14* gene and activates the expression of downstream genes, which affected the panicle branching and grain yield in rice [65, 66]. miR172 affected the flowering time and flower organ development in plants by regulating the expression of *AP2* genes [67]. Blue light alters the expression of miR167 and the target genes of auxin response factor genes in *Arabidopsis*, which finally affected the process of plant growth and generative development [68]. Research on the miRNA of *S. canadensis* shown that many DEMs were detected in flower bud and fruit development stages. Some DEMs have the same trend of expression in flower bud and fruit development stages, for example, the expression level of miR156b_2, and miR167d_1 were down-regulated in both stages of hexaploid *S. canadensis*. While, the expression level of miR156k_2, miR167d-5p and miR168a were up-regulated. Some miRNAs owing opposite trends of expression level between flower bud and fruit development stage. For example, miR156a-3p was up-regulated in flower bud stage, however, it down-regulated in fruit development stage; miR166m_2 was down-regulated in flower bud stage, however, it up-regulated in fruit development stage. There were also some miRNAs that were specifically differentially expressed in flower bud stage, such as miR162-3p, miR167f-5p and miR396b, and fruit development stage, such as miR156f, miR156f-5p, miR166-3p and miR172c-3p. The number of DEMs was different between flower bud and fruit development stages, which indicated that miRNAs may play special roles in different periods of hexaploid *S. canadensis*. Some of DEMs were predicted to target genes which involved in sexual reproduction, such as, miR156, miR156a and miR156b_2. These results suggest that DEMs may be involved in the growth, development and reproduction of *S. canadensis*.

## Conclusions

We combined transcriptome and miRNA sequence technologies to study the molecular mechanism of invasiveness in hexaploid *S. canadensis* under the sexual reproduction. In this study, based on the screening of differentially expressed genes and miRNAs in flower bud and fruit development stages of hexaploid *S. canadensis*, we found that many genes involved in the reproductive development process were up-regulated in hexaploid. In addition, some miRNAs involved in the expression regulation process may play an important role in the reproductive development of hexaploid *S. canadensis*. The result may lead to hexaploid *S. canadensis* owing higher yield and fruit quality in the process of sexual reproduction and higher germination rate of seeds which conductive to diffusion, faster propagation process and enhanced invasiveness.

## Materials and methods

### Plant materials, cDNA and small RNA library construction and sequencing

The inflorescence of hexaploid cytotype (2n = 2x = 54), which contained in flower bud (HFa) and fruit development (HFb) stages, were collected from Wuhan (30°32′N, 114°25′E), Hubei Province, China. The rhizomes of diploid cytotype (2n = 2x = 18) were transplant into the Wuhan University open-air garden from Kunming (24°55′N, 102°47′E), Yunnan Province, China [69]. The diploid were used for flower bud (DFa) and fruit development (DFb) preparation. Three replicates for each sample of hexaploid and diploid were harvested from three independent individuals. The fresh tissues between two cytotypes were collected and immediately frozen in liquid nitrogen and stored at − 80 °C for further investigation. *S. canadensis* as an invasive plant in China and the specimen has been deposited in many publicly available herbarium, such as Herbarium of Chen shan Botanical Garden (0007639), Wuhan Botanical Garden Herbarium, Chinese Academy of Sciences (0005503). The plant material used in the study was consistent with Xu et al. 2019, and the formal identification was undertaken by Xu.

Total RNAs were extracted using TRIzol Reagent and then treated with DNase I. The RNA quality was verified by Agilent 2100 Bioanalyzer (Agilent RNA 6000 Nano Kit) with RIN number > 7.0. The cDNA and small RNA library were constructed by the methods provided by Beijing Genomics Institute (BGI, Shenzhen, China).

### De novo assembly and unigene annotation

All of the constructed libraries were sequenced on an Illumina Hiseq X ten (Illumina Inc., MI, USA) platform. The raw reads of sequencing which include low-quality, contained adaptors, high content of unknown bases (more than 5%) and low-quality bases (more than 20% of the bases with a quality score less than 15) were removed. The trinity (version: v2.0.6) software with parameters as follows: --min_contig_length 150 --CPU 8 --min_kmer_cov 5 --min_glue 5 --bfly_opts’-V 5 --edge-thr = 0.1 --stderr’ [70] were used to assemble the obtained clean reads. Then the TGICL (version: v2.0.6) software with parameters as follows: -l 40 -c 10 -v 25 -O′-repeat_stringency 0.95 -minmatch 35 -minscore 35′ [71] were used to further cluster the transcripts to remove the redundant Trinity-generated transcripts. Finally, the “All-Unigene” was obtained for subsequent analysis. The “All-Unigene” sequences were aligned with the Kyoto Encyclopedia of Genes and Genomes (KEGG) public databases [72] by Blast (version: v2.2.23) and Gene Ontology (GO) by Blast2GO (version: v2.5.0) software [73] with the default parameter respectively.

### Transcription factor (TF)-encoding gene prediction

To identify candidate genes for TF, getorf (version: EMBOSS: 6.5.7.0) software with parameters as follows: -minsize 150 was used to detect the ORF of unigene [74], and then used hmmsearch (version: v3.0) with default parameter [75] to identify the ability of the TF gene family according to the characteristics described by the PlantTFDB database.

### Quantification of gene expression level and analysis of differently expressed genes

The Bowtie2 (version: v2.2.5) software [76] was used to align clean reads to assembled “All-Unigene” with the parameters as follows: -q --phred64 --sensitive --dpad 0 --gbar 99,999,999 --mp 1,1 --np 1 --score-min L,0,-0.1 -p 16 -k 200, and then based on the fragments per kilobase of transcript per million mapped reads (FPKM) analysis, RSEM (version: v1.2.8) software [77] was used to normalize the gene expression levels of each sample with the default parameter. The DEGseq [78] was used to identify differentially expressed genes (DEGs) by the value of |log_2_ Ratio| > 1.00 and adjusted *p*-value < 0.001 which was corrected by FDR for comparisons between diploid and hexaploid cytotypes with three biological replicates. Based on the result of annotation, GO and KEGG enrichment analyses were performed by phyper function. The hypergeometric test with the threshold of FDR (*Q*-value < 0.05) to find the significantly enriched terms (or pathways) in DEGs compared with the whole background.

### Weighted gene co-expression network analysis (WGCNA)

WGCNA was used in gene co-expression network identification, which has been widely applied in various biological contexts for gene expression studies, and can be used for finding highly correlated genes from clusters or modules [79]. All the DEGs of the flower bud and fruit development stage between two cytotypes were used to construct gene network by R package WGCNA. The clustered modules were collected numbers of genes, which were assembled by unique color. For each module which satisfied with correlation coefficient > 0.6 and *p* < 0.05 as the significant correlation. The co-expressed genes in these modules were extracted and visualized by Cytoscape 3.7.1 for network construction [80].

### Identification of known and novel miRNAs in *S. canadensis*

The clean reads were obtained by removing low-quality contaminated reads and adaptors. The length of clean reads which range from 18 to 30 nt was chosen for further analysis. The unique reads were immediately used to search against the miRBase 22.0 database by using the BLASTn program to annotate the conserved miRNAs. In addition, the miRNA precursor can be characterized by its hairpin structure, which was used to predict novel miRNA. Here, we used the software miRA (V1.2.0) [81] to predicted novel miRNAs through exploring the secondary structures and subsequent precursor.

### Differentially expressed miRNAs (DEMs) analysis

The expression profiles for small RNAs were calculated by using Transcripts Per Kilobase Million (TPM), which based on the formula as follows: Normalized expression = Actual miRNA count × 10^6^/Total count of clean reads. After normalization, the calculated small RNA expression level data was used directly to perform the differential expression analysis between the samples by the DEGseq R package. The threshold which satisfied with |log_2_ Ratio| > 1.00 and adjusted *p*-value < 0.001 were acted as significant DEMs.

### Target gene prediction

To find more accurate targeted genes of miRNAs, multiple types of software were used. We used psRobot (version: V1.2) software [82] with parameter as follows: -gl 17 -p 8 -gn 1 and TargerFinder (version: V1.0) software [83] with parameter as follows: -c 4 to predict miRNA targets. The function of these target genes were also detected by using similar method based on GO and KEGG database.

### MiRNA-mRNA interaction network analysis

The annotation and GO enrichment analysis of target genes was performed to explore the biological and critical functions of DEMs. Based on the integrated analysis of DEMs and the target genes, the Cytoscape 3.2.0 was used to construct the miRNA-mRNA regulatory network.

### Quantitative real-time PCR (qRT-PCR) validation

To validate the sequencing result, qRT-PCR was used to detect the expression patterns of DEGs and DEMs of each stage of diploid and hexaploid *S. canadensis*. The primer sequences of qRT-PCR were designed with Primer 5 software. The GAPDH gene was used as an internal control. The selected genes and miRNAs were calculated by the 2 ^−△△Ct^ method for relative expression with the PCR reaction condition was set as first denaturation at 95 °C in 10 min, then followed by 40 cycles of denaturation at 95 °C for 10 s, annealing and extension at 60 °C for 30 S.

## Supplementary Information

**Additional file 1.**

**Additional file 2.**

**Additional file 3.**

**Additional file 4.**

**Additional file 5.**

**Additional file 6.**

**Additional file 7.**

**Additional file 8.**

**Additional file 9.**

**Additional file 10.**

**Additional file 11.**

**Additional file 12.**

**Additional file 13.**

**Additional file 14.**

**Additional file 15.**

**Additional file 16.**

## Data Availability

All RNA-seq data associated with this study have been submitted to the NCBI Sequence Read Archive, (Accession number: PRJNA726048, https://dataview.ncbi.nlm.nih.gov/object/PRJNA726048?reviewer=j2krcdp2ntv9h5e7hhsr7e5qg5).

## Abbreviations

DFa: Flower bud stage in diploid
DFb: Fruit development in diploid
HFa: Flower bud stage in hexaploid
HFb: Fruit development in hexaploid
DEG: Differentially Expressed Gene
TF: Transcription Factor
KEGG: Kyoto Encyclopedia of Genes and Genomes
GO: Gene Ontology
TPM: Transcripts Per Kilobase Million
DEM: Differentially Expressed miRNA
qRT-PCR: Quantitative real-time PCR
