# Supplementary figures and images for: Integrated analysis of mRNA-seq and miRNA-seq reveals the advantage of polyploid Solidago canadensis in sexual reproduction
Source: BMC Plant Biol. 2021 Oct 11;21:462. doi: 10.1186/s12870-021-03240-x (PMC8504063; doi:10.1186/s12870-021-03240-x)

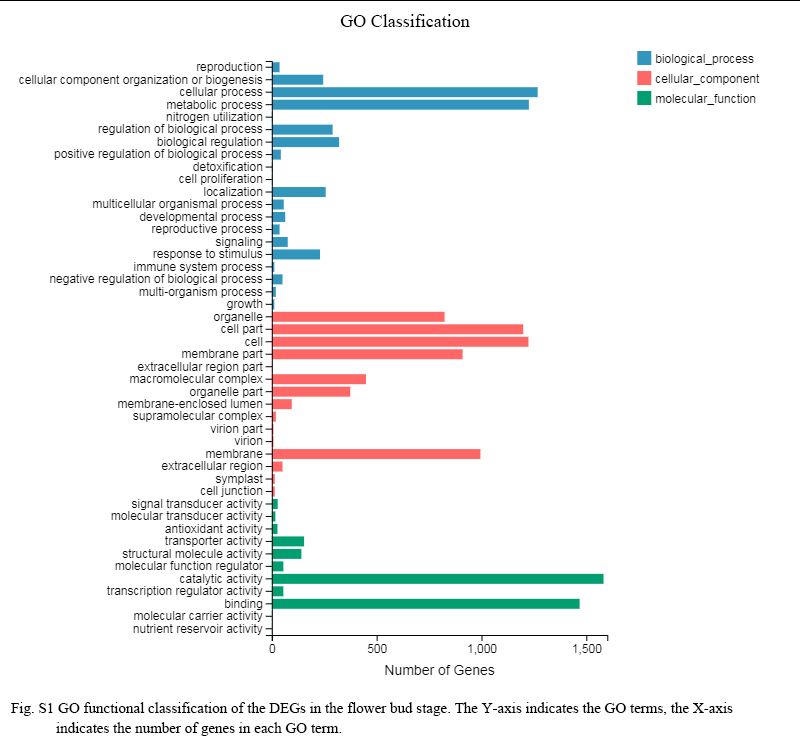

Supplement: Supplementary file 2 — Additional file 2. [file 12870_2021_3240_MOESM2_ESM.jpg]

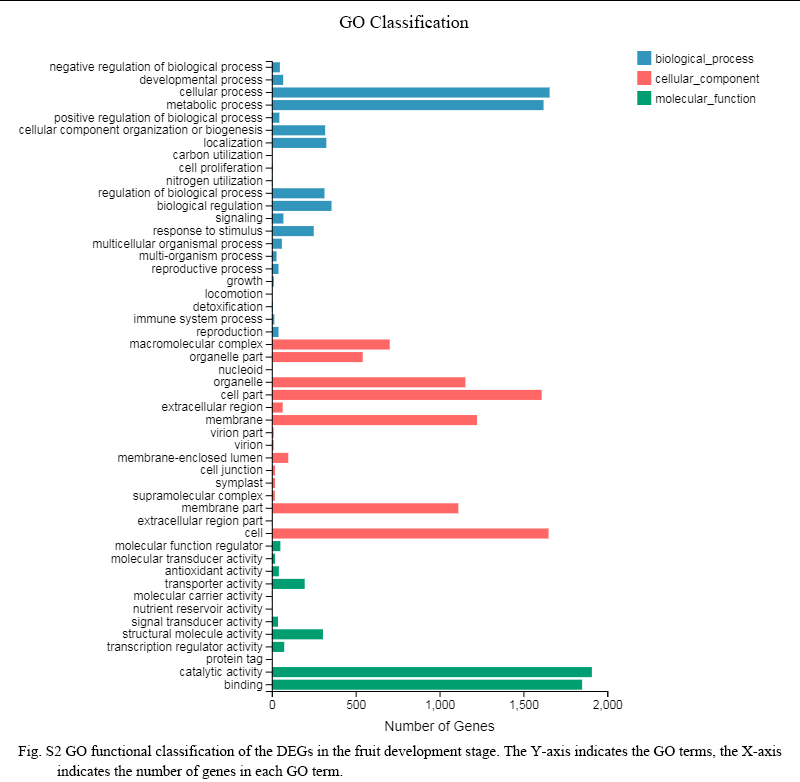

Supplement: Supplementary file 3 — Additional file 3. [file 12870_2021_3240_MOESM3_ESM.jpg]

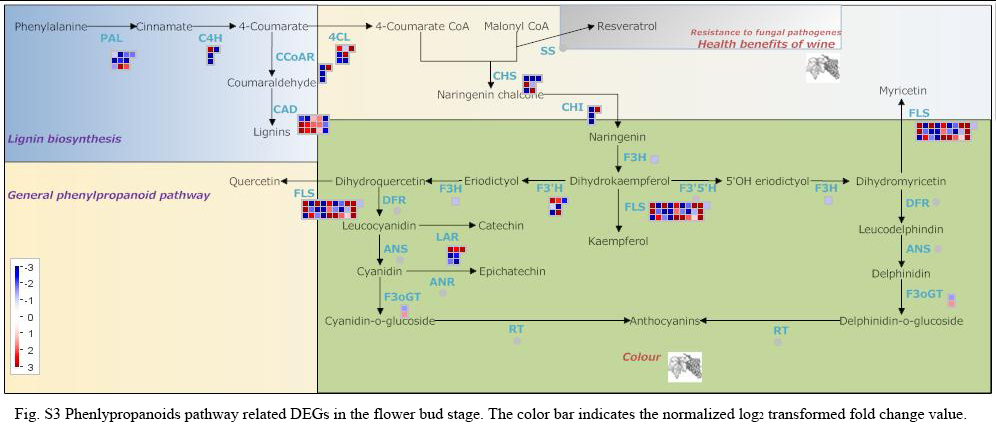

Supplement: Supplementary file 6 — Additional file 6. [file 12870_2021_3240_MOESM6_ESM.jpg]

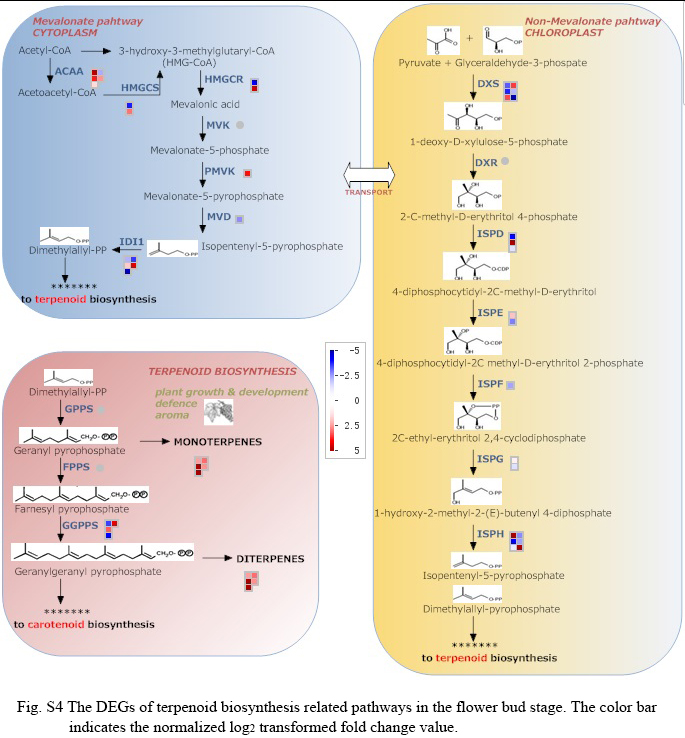

Supplement: Supplementary file 7 — Additional file 7. [file 12870_2021_3240_MOESM7_ESM.jpg]

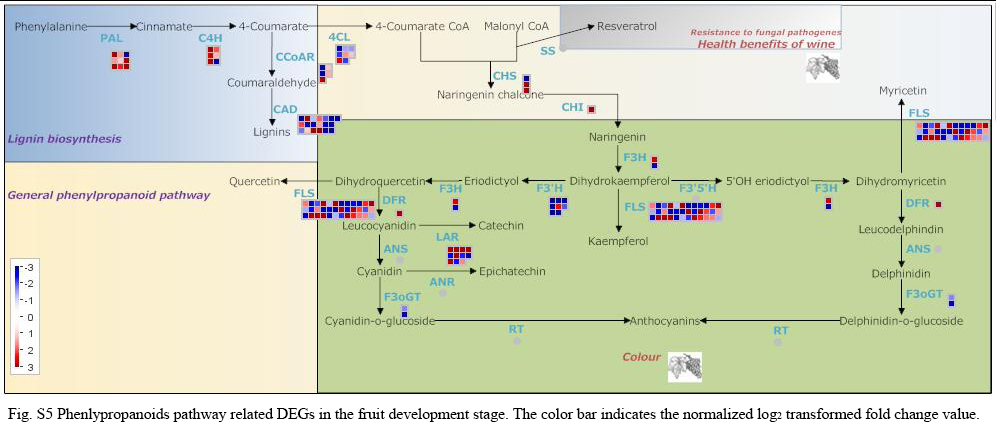

Supplement: Supplementary file 8 — Additional file 8. [file 12870_2021_3240_MOESM8_ESM.jpg]

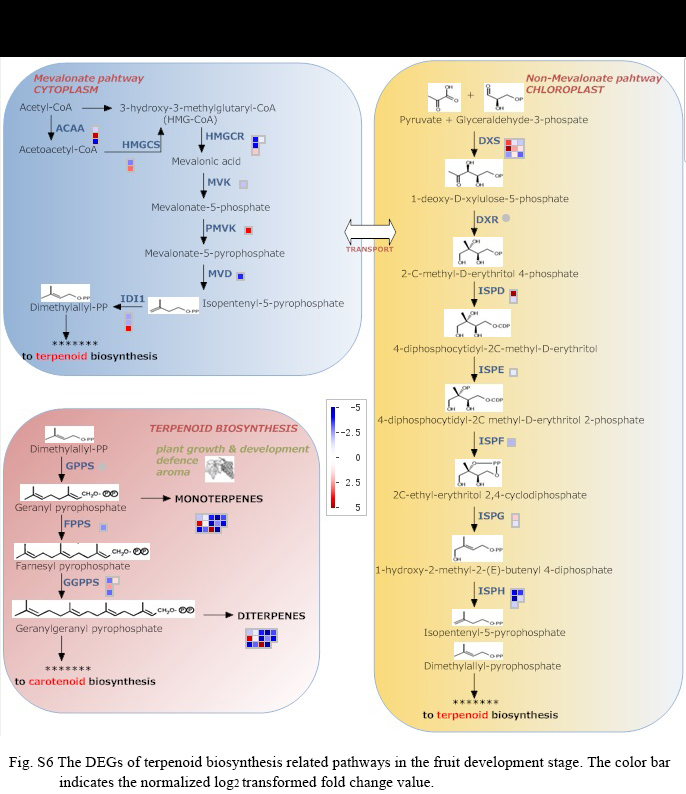

Supplement: Supplementary file 9 — Additional file 9. [file 12870_2021_3240_MOESM9_ESM.jpg]

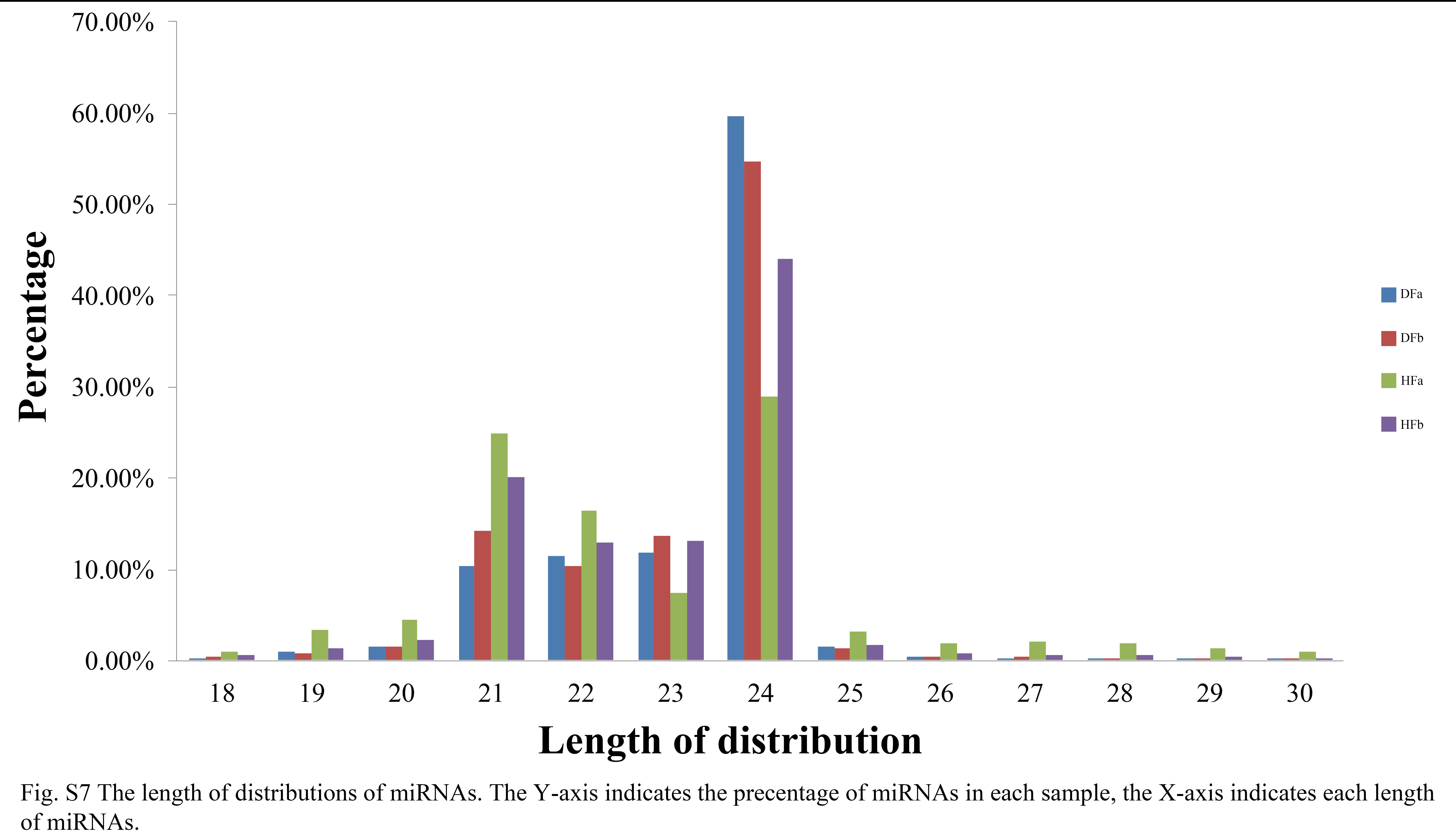

Supplement: Supplementary file 11 — Additional file 11. [file 12870_2021_3240_MOESM11_ESM.jpg]

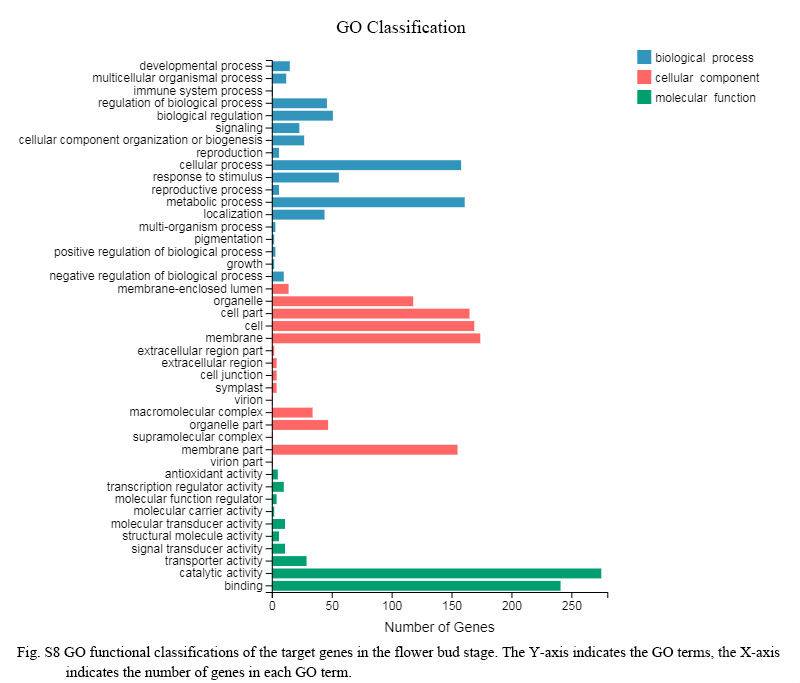

Supplement: Supplementary file 12 — Additional file 12. [file 12870_2021_3240_MOESM12_ESM.jpg]

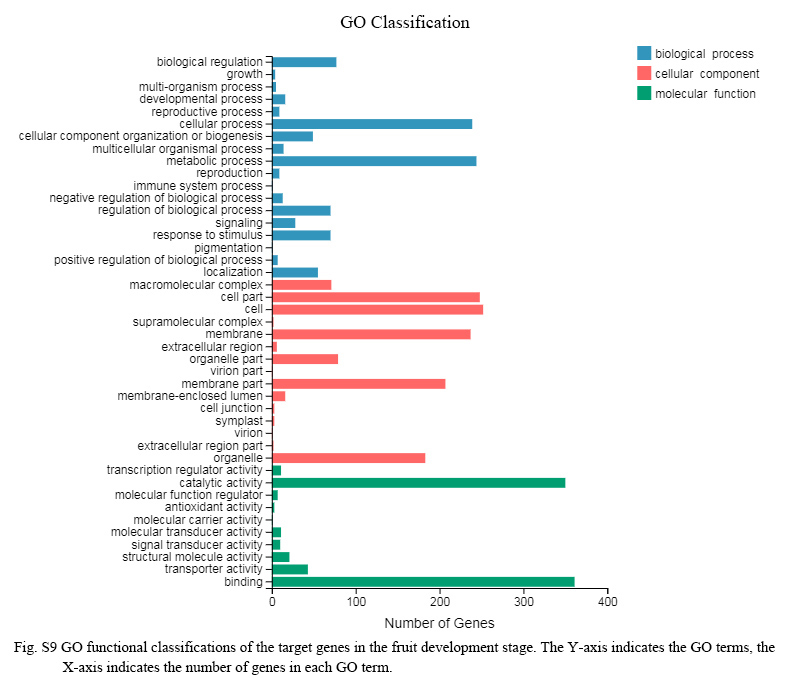

Supplement: Supplementary file 13 — Additional file 13. [file 12870_2021_3240_MOESM13_ESM.jpg]

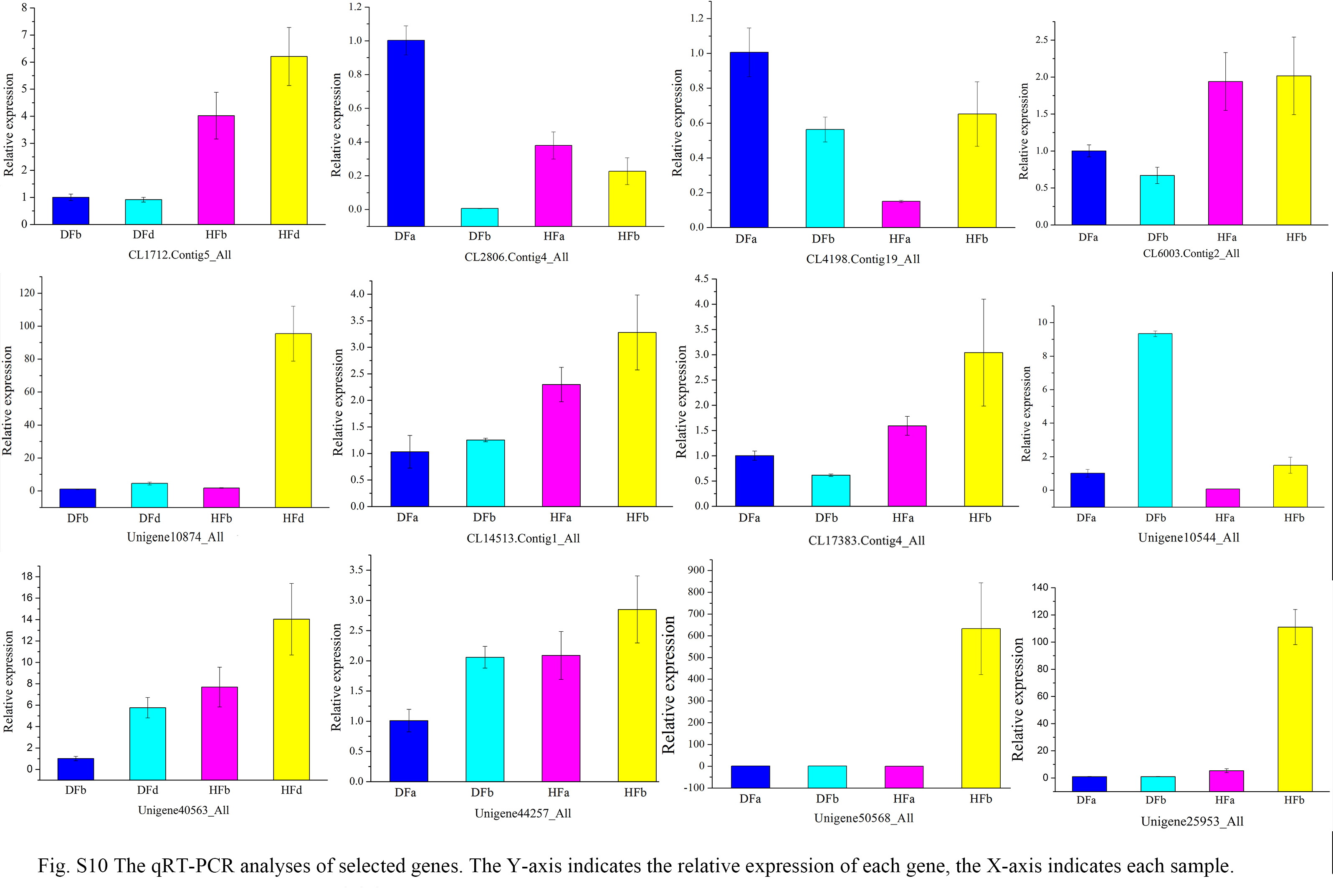

Supplement: Supplementary file 14 — Additional file 14. [file 12870_2021_3240_MOESM14_ESM.jpg]

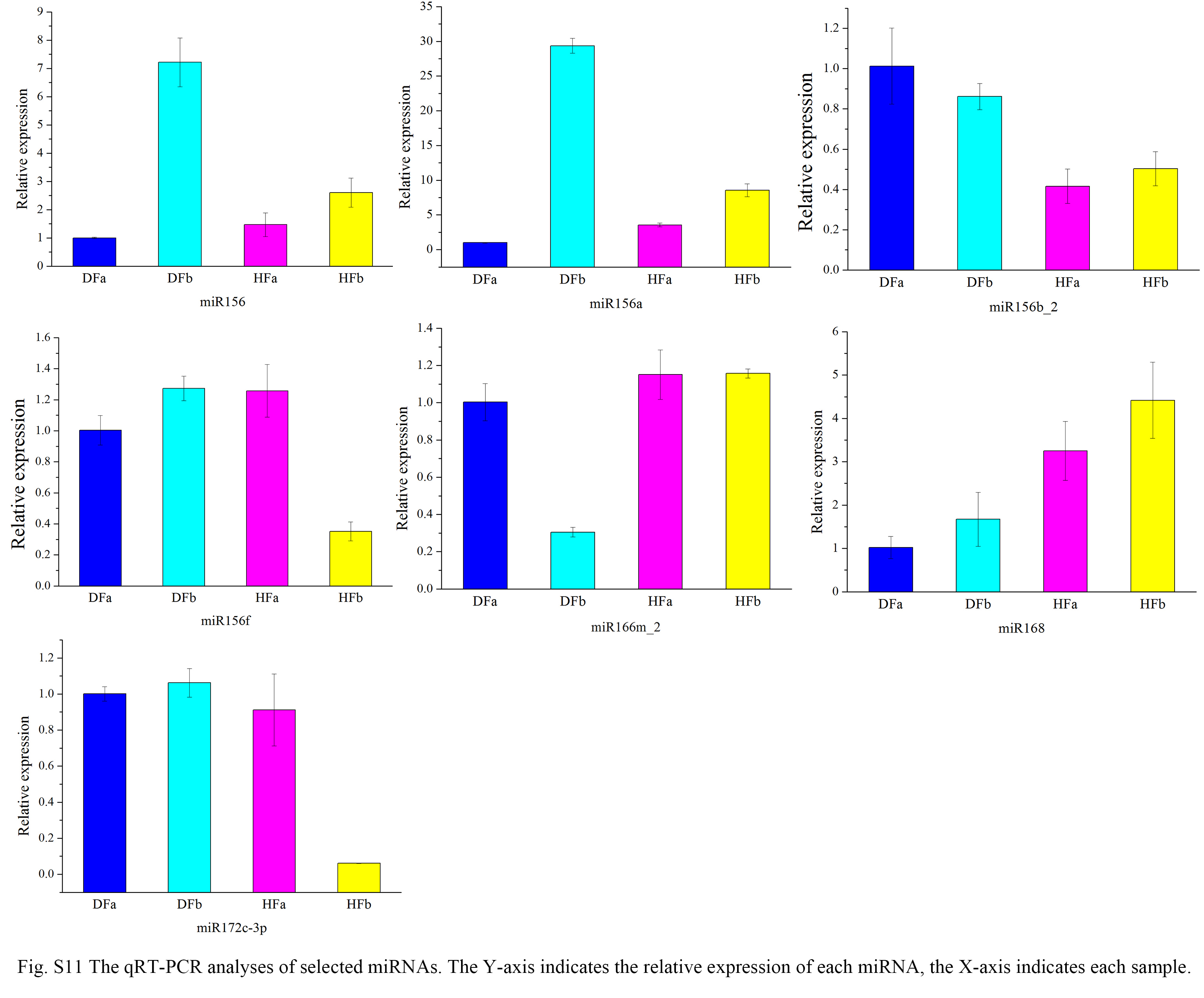

Supplement: Supplementary file 15 — Additional file 15. [file 12870_2021_3240_MOESM15_ESM.jpg]
